# Toxoplasma gondii Parasitophorous Vacuole Membrane-Associated Dense Granule Proteins Orchestrate Chronic Infection and GRA12 Underpins Resistance to Host Gamma Interferon

**DOI:** 10.1128/mBio.00589-19

**Published:** 2019-07-02

**Authors:** Barbara A. Fox, Rebekah B. Guevara, Leah M. Rommereim, Alejandra Falla, Valeria Bellini, Graciane Pètre, Camille Rak, Viviana Cantillana, Jean-François Dubremetz, Marie-France Cesbron-Delauw, Gregory A. Taylor, Corinne Mercier, David J. Bzik

**Affiliations:** a Department of Microbiology and Immunology, The Geisel School of Medicine at Dartmouth, Lebanon, New Hampshire, USA; b Laboratoire Techniques de l’Ingénierie Médicale et de la Complexité—Informatique, Mathématiques, Applications, Grenoble (TIMC-IMAG), Université Grenoble Alpes, Grenoble, France; c Centre National de la Recherche Scientifique, Unité Mixte de Recherche 5525, Grenoble, France; d Department of Medicine, Duke University Medical Center, Durham, North Carolina, USA; e Department of Molecular Genetics and Microbiology, Duke University Medical Center, Durham, North Carolina, USA; f Department of Immunology, Duke University Medical Center, Durham, North Carolina, USA; g Division of Geriatrics, Duke University Medical Center, Durham, North Carolina, USA; h Center for the Study of Aging and Human Development, Duke University Medical Center, Durham, North Carolina, USA; i Université Montpellier 2, Montpellier, France; j Centre National de la Recherche Scientifique, Unité Mixte de Recherche 5235, Montpellier, France; k Geriatric Research, Education and Clinical Center, VA Medical Center, Durham, North Carolina, USA; Albert Einstein College of Medicine

**Keywords:** *Toxoplasma gondii*, chronic infection, dense granule, innate immunity, membrane proteins, virulence factors

## Abstract

Toxoplasma gondii evades host immunity to establish a chronic infection. Here, we assessed the role of parasitophorous vacuole (PV) membrane (PVM)- and intravacuolar network (IVN) membrane-localized dense granule (GRA) proteins in the development of acute and chronic *Toxoplasma* infection. Deletion of PVM-associated GRA3, GRA7, GRA8, and GRA14 or IVN membrane-associated GRA2, GRA9, and GRA12 in the low-virulence type II Prugniaud (Pru) strain induced severe defects in the development of chronic-stage cysts *in vivo* without affecting the parasite growth rate or the ability to differentiate into cysts *in vitro*. Acute virulence of the PruΔ*gra2*, PruΔ*gra3*, and PruΔ*gra4* mutants was reduced but not abolished. In contrast, the PruΔ*gra12* mutant was avirulent in mice and PruΔ*gra12* parasites failed to establish a chronic infection. High-virulence type I strain RHΔ*gra12* parasites also exhibited a major defect in acute virulence. In gamma interferon (IFN-γ)-activated macrophages, type I RHΔ*gra12* and type II PruΔ*gra12* parasites resisted the coating of the PVM with host immunity-related GTPases as effectively as the parental type I RHΔ*ku80* and type II PruΔ*ku80* strains, respectively. Despite this resistance, Δ*gra12* PVs ultimately succumbed to IFN-γ-activated host cell innate immunity. Our findings uncover a key role for GRA12 in mediating resistance to host IFN-γ and reveal that many other IVN membrane-associated GRA proteins, as well as PVM-localized GRA proteins, play important roles in establishing chronic infection.

## Abstract (precis)

Toxoplasma gondii cysts reactivate during immune deficiency and cause fatal encephalitis. Parasite molecules that coordinate the development of acute and chronic infection are poorly characterized. Here, we show that many intravacuolar network membrane and parasitophorous vacuole membrane-associated dense granule (GRA) proteins orchestrate the development of chronic cysts *in vivo*. A subset of these GRA proteins also modulate acute virulence, and one protein that associates with the intravacuolar network membranes, namely GRA12, was identified as a major virulence factor required for parasite resistance to host gamma interferon (IFN-γ). Our results revealed that many parasitophorous vacuole membrane and intravacuolar network membrane-associated GRA proteins are essential for successful chronic infection.

## INTRODUCTION

A third of the world’s human population is currently infected by Toxoplasma gondii (1). *Toxoplasma* infection is acquired by incidental consumption of oocysts shed from cats in contaminated water or vegetables or by ingestion of tissue cysts present in undercooked infected meat (2). While immunocompetent hosts typically control the infection, *Toxoplasma* can cause severe pathology in the eye or in the developing fetus (3), particularly when the infection is triggered by a virulent strain (4). Three major strain types (type I, type II, and type III) of *Toxoplasma* predominate in North America and Europe and exhibit different virulence profiles in laboratory strains of mice: type I strains are highly virulent, type II strains exhibit low virulence, and type III strains are essentially avirulent (5).

*Toxoplasma* invades host cells and replicates within a parasitophorous vacuole (PV). Following dissemination and replication in endothelial cells at the blood brain barrier, parasites penetrate into the central nervous system (6). Primarily within neurons, tachyzoites differentiate into quiescent bradyzoites inside a modified PV termed the cyst (7, 8), which is delimited by a thick cyst wall (7). Persistence of these cysts in immune-privileged organs (9) characterizes a successful chronic infection (10, 11). If host immunity wanes, cyst reactivation causes toxoplasmic encephalitis (12, 13). The biology of cyst formation and chronic infection remains poorly understood.

*Toxoplasma* enters its host cells through an active motility-based invasion mechanism. Invasion and PV formation require temporally coordinated secretion of specialized parasite proteins from Apicomplexa-specific organelles, namely, the micronemes and the rhoptries (14). Soon after PV formation, the parasite secretes the contents of the dense granules into the nascent PV (15). The secreted dense granule (GRA and GRA-like) proteins then either remain soluble within the PV or traffic to the intravacuolar network (IVN) membranes, to the limiting PV membrane (PVM), to the host cell cytoplasm, or to the host cell nucleus (16).

GRA3, GRA7, GRA8, GRA14, and GRA15 are PVM associated (16). GRA3 is a type I PVM-associated transmembrane protein (17). GRA7 associates with ROP2 and ROP4 (18) and functions in concert (19, 20) with ROP18 protein complexes that resist gamma interferon (IFN-γ)-activated host immunity-related (p47) guanosine triphosphatases (IRGs) (21–33). ROP18 also resists host IFN-γ-activated guanylate binding proteins (GBPs) (34, 35). Recombinant GRA7 signals tumor necrosis factor (TNF) receptor-associated factor 6 (TRAF6) to activate mitogen-activated protein kinases in macrophages (36). GRA7 acts as a garroting protein that sequesters host endolysosomes within the PV (37). While GRA7 deforms liposomes into tubular membranes *in vitro* (37), Δ*gra7* parasites induce a hyperformation of the IVN membranes (38). The role of GRA8 at the PVM is currently unknown, but the protein was recently shown to play an independent role in organization of the subpellicular cytoskeleton and motility (39). GRA14 localizes to membranous extensions (PV extensions [PVE]) that radiate from the PVM into the host cell cytoplasm (40). GRA14 can also be exchanged between different PVs through PVE (40). GRA15 activates the host nuclear factor kappa-light-chain-enhancer of activated B cells (NF-κβ) pathway to drive classical activation of macrophages (41–43).

The membranes of the IVN, previously dubbed the membranous nanotubular network (MNN) (16), form a highly curved tubulovesicular network of elongated nanotubules that connect the intravacuolar tachyzoites to each other and to the PVM (44, 45). F-actin filaments of 5 nm in diameter and over 100 nm in length were recently shown to form within the membranous nanotubules of 50 to 60 nm in diameter that likely constitute the IVN (46). These F-actin filaments were shown to regulate the architecture of the IVN, the formation of the residual body, parasite organization within the PV, and the movement of vesicles between parasites within the PV (46). GRA2 (47, 48), GRA4 (49), GRA6 (50), GRA9 (51), and GRA12 (52) are IVN associated. The IVN in Δ*gra6* parasites consists of disconnected small vesicles instead of nanotubular membranes (38, 53, 54), while the IVN in Δ*gra2* parasites consists of fine aggregated material (38, 53–55). GRA2 and GRA6, through lipid binding, reshape large unilamellar vesicles into nanotubular membranes *in vitro* (53). GRA2 and GRA6 are thus key molecules that establish the characteristic nanotubular morphology of the IVN membranes of the PV. GRA6 was also recently identified as a type I strain-dependent activator of a host transcription factor, nuclear factor of activated T cells 4 (NFAT4) (56). Type I Δ*gra2* parasites exhibit a slight reduction of acute virulence in mice (54, 57), altered presentation of parasite PV antigens by host major histocompatibility complex I (MHCI) (53), and decreased antitumor T cell responses in a model of *Toxoplasma*-induced ovarian cancer immunotherapy (58, 59).

The PV ingests host membranes to satisfy the parasites’ appetite for lipids (60). These host lipids are dynamically incorporated into the IVN membranes (61). *Toxoplasma* uses PV membranes to sequester and internalize host proteins, lipid droplets, membrane vesicles, and organelles through heterophagy (37, 60, 62, 63). Δ*gra2* PVs that lack IVN nanotubular membrane structures exhibit decreased heterophagic ingestion of host cell proteins (64), Rab-positive (Rab^+^) host cell vesicles (63), and Rab7-positive (Rab7^+^) host lipid droplets (62). Moreover, acquisition of host cell cargo by heterophagy in chronic-stage cysts is also essential for successful chronic infection (65).

We previously reported that two IVN membrane-associated GRA proteins, namely, GRA4 and GRA6, are essential for successful chronic infection (66). Several PVM and IVN-associated GRA proteins have been reported to localize to the cyst wall (7, 67, 68). Δ*cst2* parasites, which fail to express the cyst wall GRA protein CST2, were recently shown to have a virulence defect, and this mutant exhibited a loss of detectable cyst burdens (68). In addition, a recent gene regulatory network analysis of *Toxoplasma* identified a small cluster of parasite genes associated with the pathogenesis community (69). This cluster includes the *GRA1*, *GRA2*, *GRA6*, and *GRA12* genes and is predicted to represent a master regulatory node common to all parasite life stages. While GRA2, GRA6, and GRA12 associate intimately with the IVN membranes as integral membrane proteins (16), GRA1 only associates weakly with the IVN through peripheral interactions (45).

We investigated 10 type II parasite mutants that lack expression of PVM-associated GRA proteins (GRA3, GRA7, GRA8, GRA14, and GRA15) or IVN-associated GRA proteins (GRA2, GRA4, GRA6, GRA9, and GRA12). Our results identified GRA12 as a key *Toxoplasma* virulence factor that resists host IFN-γ-activated innate immunity. PVM and IVN localized GRA proteins play important roles in establishing chronic infection.

## RESULTS

### Genetic deletion of PVM- and IVN-associated dense granule proteins.

We targeted the genetic deletion of IVN-associated GRA proteins (GRA2, GRA9, and GRA12) and several PVM-associated GRA proteins (GRA3, GRA7, GRA8, GRA10, GRA14, and GRA15) in the low-virulence type II Prugniaud (Pru)Δ*ku80* genetic background (66). Targeted *GRA* gene deletion (Fig. S1A in the supplemental material) was successful at each targeted gene locus, except for *GRA10* (Table S1). Absence of the targeted GRA protein was confirmed in immunofluorescence assays (IFA) (Fig. S2A).

10.1128/mBio.00589-19.1 FIG S1 GRA knockout and complementation strategies. (A) Knockout strategy to insert *HXGPRT* at deleted *GRA* gene loci by selection with mycophenolic acid (MPA) and xanthine (X). Double crossovers occurred at the *GRA* GOI locus when the PruΔ*ku80*Δ*hxgprt* strain allowed the replacement of the targeted *GRA* coding sequence with that of the *HXGPRT* resistance marker, leading to the isolation of the PruΔ*ku80*Δ*gra*(GOI)::*HXGPRT* strain. Location of the genotype validation PCRs is shown above the schematized PruΔ*ku80*Δ*gra*(GOI)::*HXGPRT* genome. (B) Complementation strategy at the *UPRT* locus through selection with 5-fluorodeoxyuridine (FUDR). A double crossover at the *UPRT* locus allowed replacement of the *UPRT* coding sequence by the *GRA12* 5′ UTR followed by the *GRA12* coding sequence; leading to the isolation of PruΔ*ku80*Δ*gra*(GOI)::*HXGPRT* Δ*uprt*::*GRA12*. Locations of the genotype validation PCRs are shown above the schematized PruΔ*ku80*Δ*gra*(GOI)::*HXGPRT* genome. Download FIG S1, TIF file, 0.5 MB. Copyright © 2019 Fox et al. 2019 Fox et al. This content is distributed under the terms of the Creative Commons Attribution 4.0 International license.

10.1128/mBio.00589-19.2 FIG S2 Validation of the GRA knockouts by IFA and immunoblotting. (A) Validation of *GRA* gene deletion in type II Δ*gra* parasite mutant strains by IFA using specific anti-GRA antibodies. (B) Validation of *GRA12* deletion in RHΔ*gra12* parasites by immunoblotting using rat anti-GRA12 polyclonal antibodies. (C) Validation of the *GRA12* deletion in RHΔ*gra12* parasites by IFA using rat anti-GRA12 polyclonal antibodies. Download FIG S2, TIF file, 1.0 MB. Copyright © 2019 Fox et al. 2019 Fox et al. This content is distributed under the terms of the Creative Commons Attribution 4.0 International license.

10.1128/mBio.00589-19.6 TABLE S1 Parasite strains used or developed in this study. Download Table S1, DOC file, 0.1 MB. Copyright © 2019 Fox et al. 2019 Fox et al. This content is distributed under the terms of the Creative Commons Attribution 4.0 International license.

### PruΔ*gra2*, PruΔ*gra3*, PruΔ*gra4*, and PruΔ*gra12* parasites exhibit significant defects in virulence.

**FIG 1:**
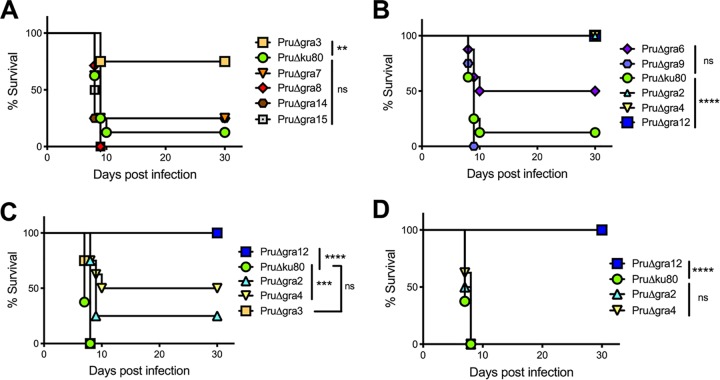
PruΔ*gra12* parasites exhibited a major defect in acute virulence. The virulence of PruΔ*gra* parasites was evaluated in mice. All data shown are from two independent experiments with 4 mice per group. The *P* values were calculated by log rank Mantel-Cox test, and a *P* value of <0.05 was considered significant. (A) C57BL/6 mice were infected intraperitoneally (i.p.) with 2 × 10^5^ tachyzoites of the PVM-associated GRA mutants PruΔ*gra3*, PruΔ*gra7*, PruΔ*gra8*, PruΔ*gra14*, and PruΔ*gra15* or the PruΔ*ku80* parent strain. (B) C57BL/6 mice were infected i.p. with 2 × 10^5^ tachyzoites of the IVN-associated GRA mutants PruΔ*gra2*, PruΔ*gra4*, PruΔ*gra6*, PruΔ*gra9*, and PruΔ*gra12* or the PruΔ*ku80* parent strain. (C) C57BL/6 mice were infected i.p. with 2 × 10^6^ tachyzoites of the PruΔ*gra2*, PruΔ*gra3*, PruΔ*gra4*, or PruΔ*gra12* parasites or the PruΔ*ku80* parental strain. (D) C57BL/6 mice were infected i.p. with 2 × 10^7^ tachyzoites of the PruΔ*gra2*, PruΔ*gra4*, or PruΔ*gra12* parasites or the PruΔ*ku80* parental strain. **, *P < *0.01; ***, *P < *0.001; ****, *P* < 0.0001; ns, not significant.

Acute-virulence phenotypes of each PruΔ*gra* mutant were examined by challenging mice intraperitoneally (i.p.) with different parasite doses. Several parasite mutants with deletion mutations of PVM-localized (PruΔ*gra3*) (Fig. 1A) or IVN-localized (PruΔ*gra2*, PruΔ*gra4*, and PruΔ*gra12*) GRA proteins (Fig. 1B) exhibited significant virulence defects after infection with 2 × 10^5^ tachyzoites. While PruΔ*gra6* parasites trended toward a reduced virulence (Fig. 1B), this difference (*P = *0.09) did not achieve statistical significance. However, only PruΔ*gra2*, PruΔ*gra4*, and PruΔ*gra12* parasites exhibited significant virulence defects after infection with 2 × 10^6^ tachyzoites, whereas PruΔ*gra3* parasites (Fig. 1C) and, as expected, PruΔ*gra6*, PruΔ*gra7*, PruΔ*gra8*, PruΔ*gra9*, PruΔ*gra14*, and PruΔ*gra15* parasites did not exhibit any virulence defect after this challenge dose (Fig. S3B). The virulence defect of PruΔ*gra4* parasites was rescued by complementation with the wild-type *GRA4* gene (Fig. S3C). Only PruΔ*gra12* parasites exhibited a significant virulence defect after infection with 2 × 10^7^ tachyzoites (Fig. 1D). No significant defect was observed in the *in vitro* replication rate of any type II PruΔ*gra* mutant (Fig. S4), which confirmed the results previously reported for both the PruΔ*gra4* and PruΔ*gra6* mutants (66). Finally, while moderate virulence defects were associated with PruΔ*gra2*, PruΔ*gra3*, and PruΔ*gra4* parasites, PruΔ*gra12* parasites were, surprisingly, avirulent.

10.1128/mBio.00589-19.3 FIG S3 Virulence of Δ*gra* parasites and rescue of Δ*gra4* virulence. (A) PruΔ*gra4* and PruΔ*gra6* parasites differentiate to GFP^+^ cysts *in vitro* at high pH. (B) C57BL/6 mice were infected i.p. with 2 × 10^6^ tachyzoites of various PruΔ*gra* parasite strains, and virulence was measured. (C) The *GRA4* gene rescues PruΔ*gra4* virulence. ***, *P < *0.005. Download FIG S3, TIF file, 0.4 MB. Copyright © 2019 Fox et al. 2019 Fox et al. This content is distributed under the terms of the Creative Commons Attribution 4.0 International license.

10.1128/mBio.00589-19.4 FIG S4 Replication rates. Replication rates of PruΔ*gra* parasites were measured in infected HFF cells in a 45-h assay (see Materials and Methods). Download FIG S4, TIF file, 0.2 MB. Copyright © 2019 Fox et al. 2019 Fox et al. This content is distributed under the terms of the Creative Commons Attribution 4.0 International license.

### PVM- and IVN-associated dense granule proteins are essential for the development of chronic cyst burdens.

**FIG 2:**
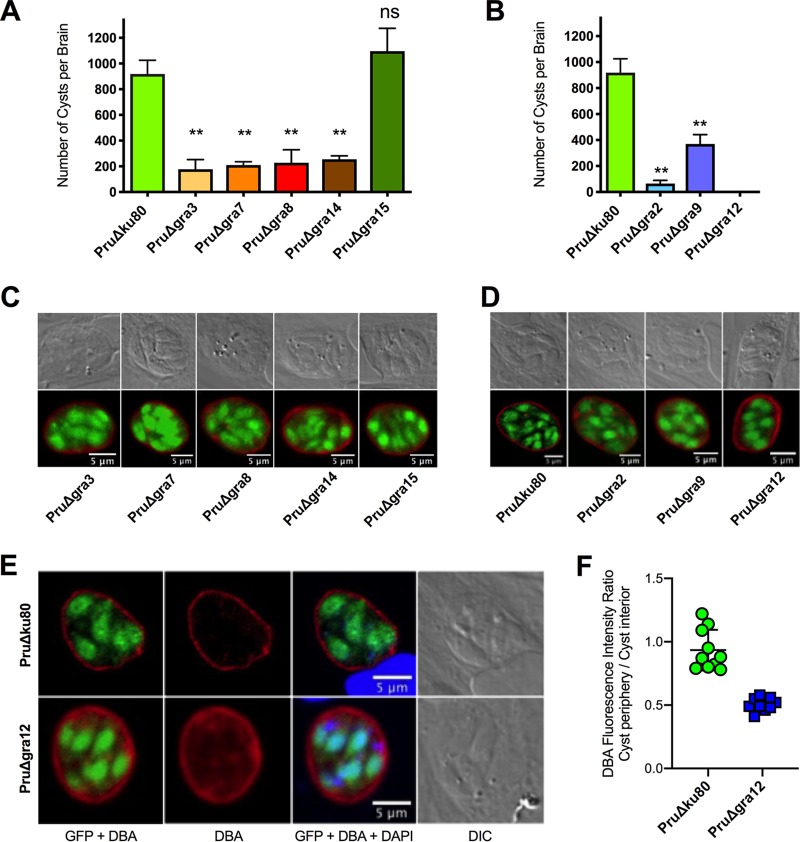
Cyst burdens were markedly reduced or abolished in parasites that lack expression of PVM- or IVN-associated GRA proteins. (A, B) C57BL/6 mice were infected i.p. with 200 tachyzoites of GRA knockout strains, and brain cyst burdens were counted 21 days after infection. The data presented are the cumulative results from 1 to 3 independent experiments for each strain tested and are shown as the mean values plus or minus standard errors of the means (± SEM). The *P* values were calculated with Student’s *t* test, with a *P* value of <0.05 considered significant. **, *P < *0.001; ns, not significant. (A) PVM-associated GRA mutants PruΔ*gra3* (1 experiment; *n = *4 mice), PruΔ*gra7* (1 experiment; *n = *4 mice), PruΔ*gra8* (1 experiment; *n = *4 mice), PruΔ*gra14* (1 experiment; *n = *4 mice), and PruΔ*gra15* (3 experiments; *n = *10 mice) and the PruΔ*ku80* parent strain (3 experiments; *n = *12 mice). (B) IVN-associated GRA mutants PruΔ*gra2* (1 experiment; *n = *4 mice), PruΔ*gra9* (2 experiments; *n = *5 mice), and PruΔ*gra12* (2 experiments; *n = *8 mice) and the PruΔ*ku80* parent strain (3 experiments; *n = *12 mice). (C, D) HFF cells were infected with the designated PVM- or IVN-associated GRA knockout strain. Infected host cells were treated under bradyzoite-inducing conditions (pH 8.1 and CO_2_ depletion in ambient air) for 3 days. The cyst wall was stained with Dolichos biflorus agglutinin coupled to Texas red (DBA) (shown in red). Bradyzoites were visualized by their GFP expression (shown in green), which is under the control of the bradyzoite stage-specific *LDH2* promoter. Samples were imaged by confocal microscopy, and PVs were located using differential interference contrast (DIC) microscopy. Representative results are shown for each strain. (E) Comparison of PruΔ*gra12* and PruΔ*ku80* cysts showing images for GFP plus DBA, DBA only, GFP plus DBA plus DAPI, and DIC. Cysts shown in this panel were cysts that scored at the mean values shown in panel F. (F) DBA fluorescence intensity was measured as the ratio of DBA fluorescence intensity at the cyst periphery compared to that in the cyst interior for PruΔ*gra12* cysts versus parental strain PruΔ*ku80* cysts. Each cyst measurement is shown in the graph. ****, *P < *0.0001.

Each PruΔ*gra* mutant was tested for its ability to establish a chronic infection in mice. Major defects in the development of brain cyst burdens were observed in the PruΔ*gra* mutants that had abrogated expression of PVM-associated (PruΔ*gra3*, PruΔ*gra7*, PruΔ*gra8*, and PruΔ*gra14*) (Fig. 2A) or IVN-associated (PruΔ*gra2*, PruΔ*gra9*, and PruΔ*gra12*) proteins (Fig. 2B). In contrast, cyst burdens were normal in PruΔ*gra15* parasites (Fig. 2A). Cyst burdens were reduced by more than 90% in PruΔ*gra2* parasite infections, similar to the >90% cyst reductions we previously reported in PruΔ*gra4* and PruΔ*gra6* parasite infections (66). In contrast, cyst burdens were reduced by ∼60% to 80% in PruΔ*gra3*, PruΔ*gra7*, PruΔ*gra8*, PruΔ*gra9*, and PruΔ*gra14* parasite infections. Remarkably, cysts were not detected in mice infected with PruΔ*gra12* parasites (Fig. 2B). Major defects in cyst burdens were associated with virulence defects in the PruΔ*gra2*, PruΔ*gra3*, PruΔ*gra4*, and PruΔ*gra12* mutants but not in the PruΔ*gra6*, PruΔ*gra7*, PruΔ*gra8*, PruΔ*gra9*, and PruΔ*gra14* mutants.

While reduced virulence can potentially explain reduced cyst burdens, reduced cyst burdens can also arise from a failure to build a resistant cyst wall or from a deficiency in tachyzoite-to-bradyzoite stage differentiation. Consequently, we examined the ability of the PruΔ*gra* mutant tachyzoites to differentiate into chronic-stage bradyzoites inside a cyst wall after *in vitro* exposure to alkaline switch conditions. After alkaline switching, all of the PruΔ*gra* mutants and the parental PruΔ*ku80* strain differentiated into bradyzoites expressing green fluorescent protein (GFP) under the control of the bradyzoite-specific *LDH2* promoter. These GFP-positive (GFP^+^) bradyzoites were visible within an apparently intact cyst wall structure revealed with fluorescently labeled Dolichos biflorus agglutinin (DBA) (Fig. 2C and D, Fig. S3A) (70).

The Golgi complex-associated aspartyl protease 5 (ASP5) is required for processing and targeting of many secreted GRA proteins. Since genetic deletion of ASP5 in type II parasites (PruΔ*asp5*) severely impairs cyst wall development (71), we further evaluated the pattern of DBA staining in PruΔ*gra12* cysts. DBA almost exclusively targets and stains the major cyst wall protein CST1, which is modified by *N*-acetylgalactosamine residues that are specifically recognized and bound by DBA (8). Relative to the results for PruΔ*ku80* cysts, PruΔ*gra12* cysts appeared to exhibit increased intensity of DBA staining inside the cyst (Fig. 2E). To quantify this observation, we randomly selected 12 PruΔ*gra12* and 10 PruΔ*ku80* 3-day-old *in vitro* cysts and measured the ratio of DBA fluorescence intensity at the cyst periphery, reflecting CST1 cargo delivered to the cyst wall, compared to the fluorescence intensity detected in the cyst interior, which highlights CST1 cargo not yet delivered to the cyst wall. In comparison to the parental PruΔ*ku80* strain that expressed GRA12, PruΔ*gra12* cysts exhibited a significant decrease in the DBA fluorescence intensity ratio (cyst periphery/cyst interior) (Fig. 2F), suggesting that GRA12 could be involved in the delivery of CST1 to the cyst wall.

### GRA12 localizes at the IVN.

**FIG 3:**
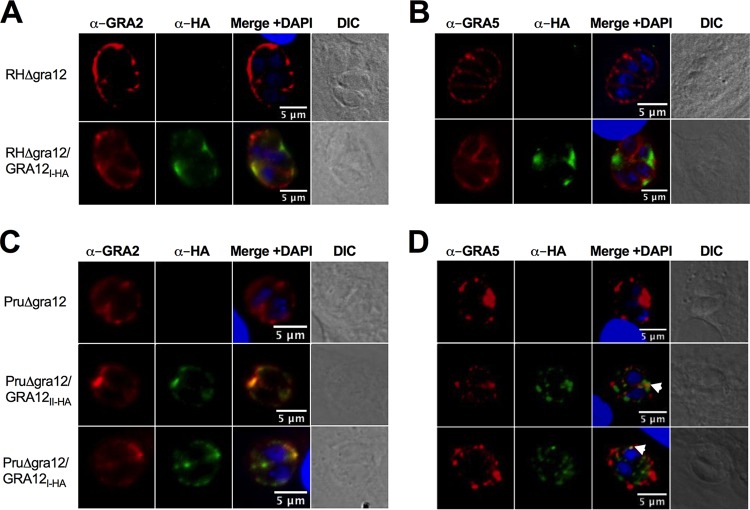
GRA12 in the acute-stage tachyzoite PV predominantly colocalizes with IVN-associated GRA2. (A to D) HFF cells were infected for 24 h and fixed with paraformaldehyde, and the PVM was permeabilized with 0.01% saponin. Antibodies specific to the HA tag positioned at the C terminus of GRA12 were used to localize GRA12 (shown in green) in the PV relative to the IVN-associated marker GRA2 (detected by monoclonal antibodies to GRA2 and shown in red) (A, C) or the PVM-associated GRA5 (detected by monoclonal antibodies to GRA5 and shown in red) (B, D). Host cell and parasite nuclei were stained with DAPI (blue). The location of PVs in the infected host cell was confirmed by differential interference contrast (DIC) microscopy. White arrowheads indicate isolated points of colocalization of GRA12 and GRA5 near the periphery of the PV. Representative images are shown.

In the virulent type I RH strain, GRA12 predominantly colocalized with GRA2 at the IVN membranes (52). To confirm this GRA12 localization, we also deleted the *GRA12* gene from the highly virulent type I RHΔ*ku80* strain (Table S1, Fig. S2B and C) (72). The GRA12 protein is highly conserved between type I, type II, and type III strains (Fig. S5). The type I and type II Δ*gra12* mutants were complemented with the type I RH strain *GRA12* gene (TGGT1_288650), thus generating the strains RHΔ*gra12/GRA12_I-HA_* and PruΔ*gra12/GRA12_I-HA_*, respectively, while type II PruΔ*gra12* parasites were complemented with the type II Pru *GRA12* gene (TGME49_288650), generating the strain PruΔ*gra12/GRA12_II-HA_*. All of these *GRA12* complementing genes were tagged with the hemagglutinin (HA) epitope placed in-frame at the C terminus of GRA12 (Fig. S1B, Table S1) to permit the visualization of GRA12 by immunofluorescence and immunoblot experiments using antibodies specific to the HA tag. As expected, GRA12 predominantly colocalized with GRA2 and the IVN membranes in type I RH (Fig. 3A) and type II Pru strains (Fig. 3C). GRA12 also sporadically colocalized with the PVM-specific marker GRA5 (Fig. 3D; white arrowheads). Sporadic colocalization of GRA12 with the PVM-specific marker GRA3 was also previously reported (52). It is not surprising that a predominantly IVN-localized protein like GRA12 could occasionally localize with PVM-localized proteins because, within the PV, the IVN membranes have been shown to form direct connections with the PVM (45).

10.1128/mBio.00589-19.5 FIG S5 Amino acid alignments of GRA12 expressed by type I (GT1), type II (ME49), and type III (VEG) strains. Protein alignments and amino acid similarity and identity scores are shown for GRA12 expressed by the type I strain (TGGT_288650), type II strain ME49 (TGME49_288650), and type III strain VEG (TGVEG_288650). Download FIG S5, DOCX file, 0.1 MB. Copyright © 2019 Fox et al. 2019 Fox et al. This content is distributed under the terms of the Creative Commons Attribution 4.0 International license.

### The mature IVN forms in the absence of GRA12 expression.

**FIG 4:**
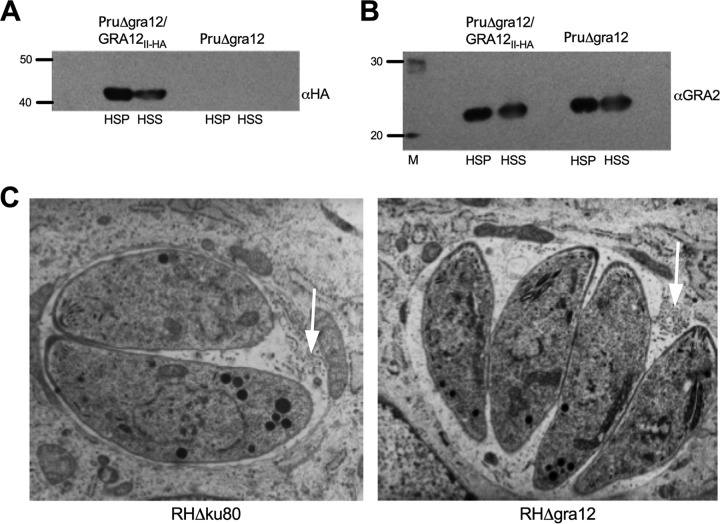
Intravacuolar network membranes form in the absence of GRA12 expression. (A) To confirm IVN membrane localization of GRA12, HFF cells containing mature PruΔ*gra12/GRA12_II-HA_* PV or mature PruΔ*gra12* PV were fractionated into a high-speed soluble fraction (HSS) and a PV membrane-associated high-speed pellet (HSP) 24 h after infection. Proteins in the HSP or the HSS fractions were separated on a 10% SDS-PAGE gel under reducing conditions in parallel to a protein molecular-weight ladder (40- and 50-kDa positions are shown), transferred to nitrocellulose membranes, incubated with rabbit anti-HA tag antibodies specific to GRA12_II-HA_, revealed with goat anti-rabbit IgG coupled to peroxidase, and visualized by chemiluminescence. (B) To assess IVN membrane localization of GRA2 in the absence of GRA12 expression, HFF cells containing the fully developed PruΔ*gra12/GRA12_II-HA_* PV or PruΔ*gra12* PV were fractionated into a high-speed soluble fraction (HSS) and a PV membrane-associated high-speed pellet (HSP) 24 h after infection as described in the legend to panel A. Western blots were incubated with anti-GRA2 mouse monoclonal antibodies, revealed with anti-mouse IgG coupled to peroxidase, and visualized by chemiluminescence. Twenty- and 30-kDa markers are shown. (C) Transmission electron microscopy of the type I RHΔ*gra12* mutant revealed the presence of a normal mature IVN (white arrows) and no tachyzoite or PV abnormalities.

We confirmed that GRA12 is an IVN-associated protein using biochemical fractionation of human foreskin fibroblast (HFF) cells that had been infected for 24 h. Typical of IVN membrane-localized GRA proteins, GRA12 partitioned into the IVN membrane fraction (high-speed pellet [HSP]) and the vacuole soluble fraction (high-speed supernatant [HSS]) (Fig. 4A). GRA2 (38, 53–55) and GRA6 (38, 53, 54) organize the nanotubular morphology of the IVN membranes. Since GRA12 colocalizes with GRA2 and GRA6 when the IVN membranes are being organized within the PV space soon after dense granule secretion, it was previously proposed by Michelin et al. that GRA12 could also play a role in organizing the nanotubular morphology of the IVN membranes (52). To determine whether other IVN GRA proteins normally localized to the IVN in the PV in the absence of GRA12 expression, we examined the biochemical fractionation of GRA2 into the HSP (IVN membrane) and HSS (PV soluble) fractions. Fractionation of HFF cells infected by Δ*gra12* parasites revealed that GRA2 still partitioned normally between the HSP and HSS fractions of the PV (Fig. 4B). In addition, using transmission electron microscopy (TEM) imaging, we did not detect any significant Δ*gra12* tachyzoite abnormality. Furthermore, we observed typical PVs decorated with normal-looking IVN membranes in Δ*gra12* PVs (Fig. 4C). Collectively, these results suggested that GRA12 was not necessary for the shaping of mature IVN membranes.

### GRA12 expression is essential for type II strain resistance to IFN-γ.

**FIG 5:**
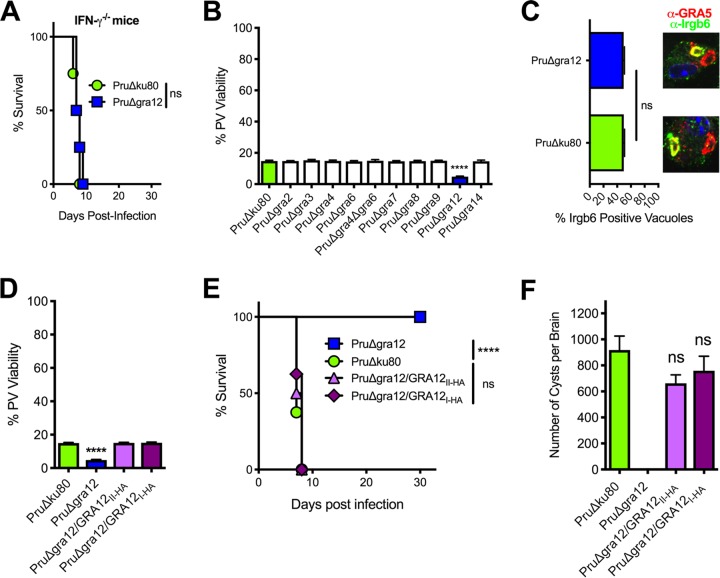
Type II PruΔ*gra12* parasites exhibit a loss of PV viability in IFN-γ-activated host cells. (A) Survival of C57BL/6 IFN-γ^−/−^ knockout mice infected intraperitoneally with 2 × 10^2^ tachyzoites of PruΔ*gra12* or PruΔ*ku80*. ns, not significant. (B) Mouse embryonic fibroblasts (MEFs) were stimulated with IFN-γ, and PV viability (measured as PFU) was determined in comparison to the results for nonstimulated (no IFN-γ) MEFs. The results from at least 3 independent experiments are shown as mean values plus or minus standard errors of the means (± SEM). Significant *P* values were calculated with Student’s *t* test (****, *P < *0.0001). (C) Quantification of Irgb6 coating of PVs 45 min after infection of IFN-γ-stimulated bone marrow-derived macrophages (BMDM) by PruΔ*gra12* or PruΔ*ku80* parasites. Representative images of PVs stained with anti-Irgb6 antibody (green) and anti-GRA5 antibody (red) are shown above the graph. At least 500 PVs were scored to determine the significance. Significant *P* values were calculated with Student’s *t* test (ns, not significant). (D) MEFs were stimulated *in vitro* with IFN-γ and infected with PruΔ*gra12* or PruΔ*ku80* mutant parasites or complemented PruΔ*gra12/GRA12_I-HA_* or PruΔ*gra12/GRA12_II-HA_* parasites. PV viability (measured as PFU) was determined in comparison to the results for nonstimulated (no IFN-γ) MEFs. Results from at least 4 independent experiments are shown as mean values ± SEM. Significant *P* values were calculated with Student’s *t* test (****, *P < *0.0001). (E) Survival of C57BL/6 mice infected i.p. with 2 × 10^6^ tachyzoites of PruΔ*gra12*, parental PruΔ*ku80*, or complemented PruΔ*gra12/GRA12_I-HA_* or PruΔ*gra12/GRA12_II-HA_* strains. The data presented are the combined results of 2 independent experiments, each with 4 mice per group. The *P* value was calculated by log rank Mantel-Cox test, and a *P* value of <0.05 was considered significant (****, *P < *0.0001; ns, not significant). (F) CD1 mice were infected i.p. with 2 × 10^2^ tachyzoites of each designated strain, and chronic-stage cyst burdens were counted 21 days postinfection. The data are cumulative results from 2 to 3 independent experiments for each strain tested, as follows: PruΔ*ku80* (3 experiments; *n *=* *12 mice), PruΔ*gra12* (2 experiments; *n *=* *8 mice), PruΔ*gra12/GRA12_I-HA_* (2 experiments; *n *=* *8 mice), and PruΔ*gra12/GRA12_II-HA_* (2 experiments; *n *=* *8 mice). ns, not significant.

The cyst burden and acute-virulence phenotypes of PruΔ*gra12* parasites closely resembled phenotypes we previously reported for PruΔ*rop5* and PruΔ*rop18* mutants (27). We initially evaluated whether type II PruΔ*gra12* parasites were virulence attenuated in mice deficient in the production of IFN-γ. IFN-γ^−/−^ knockout mice succumbed to a low infection dose of PruΔ*gra12* parasites, with kinetics similar to those in IFN-γ^−/−^ mice infected by parental type II PruΔ*ku80* parasites (Fig. 5A), suggesting that IFN-γ was required to control PruΔ*gra12* infection. We next evaluated the PV viability of all the type II PruΔ*gra* mutants compared to that of the parental PruΔ*ku80* strain in IFN-γ-activated host cells. Compared with the results for the parental PruΔ*ku80* strain, the only PruΔ*gra* mutant that exhibited an enhanced loss of PV viability in IFN-γ-activated mouse embryonic fibroblasts (MEFs) was PruΔ*gra12* (Fig. 5B).

PruΔ*rop5* and PruΔ*rop18* PVs exhibit a loss of PV viability in IFN-γ-activated murine host cells due to a failure to resist increased coating of the PVM by IFN-γ-inducible effector immunity-related GTPase (IRG) proteins (such as Irgb6) and subsequent rupture of the PV (27). We measured PVM coating by the pioneer IRG, Irgb6 (73). Irgb6 coating of the type II strain PruΔ*gra12* PVM was not increased in IFN-γ-activated murine bone marrow-derived macrophages (BMDM) (Fig. 5C). In addition, type II PruΔ*gra12* PV viability in IFN-γ-activated host cells (Fig. 5D) and the acute-virulence phenotypes (Fig. 5E) were rescued by both the type II (strain PruΔ*gra12/GRA12_II-HA_*) and the type I (strain PruΔ*gra12/GRA12_I-HA_*) *GRA12* genes. Correspondingly, normal levels of cyst burdens were also rescued in GRA12-complemented type II PruΔ*gra12* strains (Fig. 5F), suggesting that GRA12 could be a significant virulence factor that mediates resistance to host IFN-γ in both low- and high-virulence strains.

### GRA12 expression is essential for type I strain resistance to host IFN-γ.

**FIG 6:**
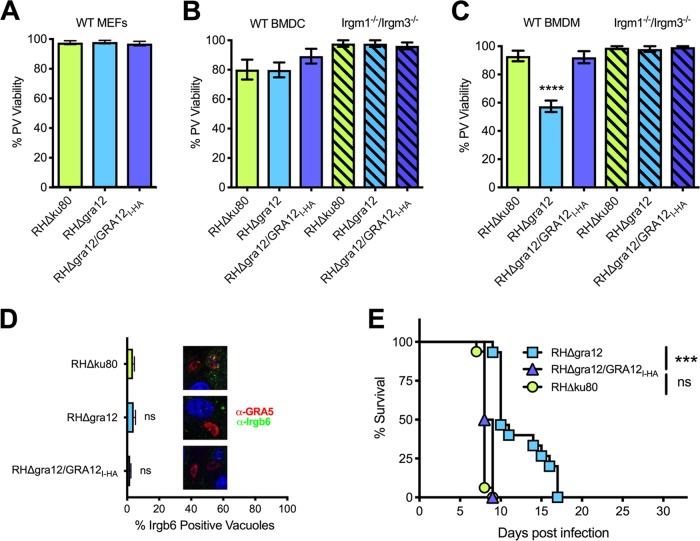
Type I RHΔ*gra12* parasites exhibit a loss of PV viability in IFN-γ-activated macrophages. (A) MEFs were stimulated with IFN-γ, and PV viability (measured as PFU) was determined in comparison to the results for nonstimulated (no IFN-γ) MEFs. The results from at least 3 independent experiments are shown as mean values plus or minus standard errors of the means (± SEM). Significant *P* values were calculated with Student’s *t* test. (B) Bone marrow-derived dendritic cells (BMDC) from wild-type C57BL/6 mice or *Irgm1*^−/−^/*Irgm3*^−/−^ knockout C57BL/6 mice were stimulated with IFN-γ, and PV viability (measured as PFU) was determined in comparison to the results for nonstimulated (no IFN-γ) BMDC. Results from at least 4 independent experiments are shown as mean values ± SEM. Significant *P* values were calculated with Student’s *t* test. (C) Bone marrow-derived macrophages (BMDM) from wild-type C57BL/6 mice or *Irgm1^−^*^/−^*/Irgm3*^−/−^ knockout C57BL/6 mice were stimulated with IFN-γ and PV viability (measured as PFU) was determined in comparison to the results for nonstimulated (no IFN-γ) BMDM. Results from at least 4 independent experiments are shown as mean values ± SEM. Significant *P* values were calculated with Student’s *t* test (****, *P < *0.0001). (D) Quantification of PV coating by Irgb6 45 min after infection of IFN-γ-stimulated bone marrow-derived macrophages (BMDM). At least 500 PVs were observed to determine significance. *P* values were calculated with Student’s *t* test. On the right side of the graph, representative images of PVs incubated with anti-Irgb6 (green) and anti-GRA5 (red) antibodies are shown. ns, not significant. (E) CD1 mice were infected i.p. with 1 × 10^2^ tachyzoites of RHΔ*gra12*, parental RHΔ*ku80*, or complemented RHΔ*gra12/GRA12_I_*. The survival data shown are from 2 independent experiments, each with 4 mice per group. The *P* value was calculated using the log rank Mantel-Cox test, and a *P* value of <0.05 was considered significant (***, *P < *0.001; ns, not significant).

We next examined the PV viability phenotype associated with the expression of GRA12 in the virulent type I RH strain. In contrast to type II PruΔ*gra12* PVs that lost PV viability in MEFs (Fig. 5D), PV viability was not affected in IFN-γ-activated MEFs (Fig. 6A) or in IFN-γ-activated bone marrow-derived dendritic cells (BMDC) (Fig. 6B) infected with type I RHΔ*gra12* parasites. In contrast, PV viability was significantly decreased in IFN-γ-activated murine BMDM infected with RHΔ*gra12* parasites, and this macrophage-specific loss of PV viability was rescued by complementation with a type I RH strain *GRA12* gene (strain RHΔ*gra12/GRA12_I-HA_*) (Fig. 6C). The loss of PV viability of type I RHΔ*gra12* parasites in IFN-γ-activated BMDM was dependent on host IRGM1 and IRGM3, since a decrease in PV viability was not observed in macrophages derived from *Irgm1*^−/−^/*Irgm3*^−/−^ double knockout mice (Fig. 6C) (74, 75) . In addition, PV resistance to Irgb6 PVM coating was not affected in IFN-γ-activated BMDM infected with RHΔ*gra12* parasites (Fig. 6D). A major virulence defect was observed after infection of CD1 mice with type I RHΔ*gra12* parasites, and this virulence defect was rescued by complementation with the type I *GRA12* gene (Fig. 6E). Thus, in both low- and high-virulence parasite strain types, GRA12 determined the ability of the PV to resist host IFN-γ.

## DISCUSSION

The presence of cysts in the central nervous system characterizes the chronic stage of *Toxoplasma* infection (6, 10, 11). A limiting membrane termed the cyst membrane surrounds the cyst wall (7, 8), and while this cyst membrane is hypothesized to arise from the modified PVM (7, 8, 76), the fate and trafficking of PVM and IVN membrane proteins during differentiation and cyst development still remain to be investigated. Similar to the IVN membranes present in acute-stage tachyzoite PVs, mature cysts contain membrane tubules that, within an intracyst matrix, link the bradyzoites to each other and to the cyst wall and, possibly, to the cyst membrane (7). Our study showed that all the IVN-associated GRA proteins (GRA2, GRA4, GRA6, GRA9, and GRA12) and all the PVM-associated GRA proteins (GRA3, GRA7, GRA8, and GRA14) we investigated, except GRA15, were crucial for successful chronic infection.

PruΔ*gra* parasite mutants retained a normal replication rate and differentiated to establish GFP^+^ bradyzoites encased by a cyst wall. In contrast to PruΔ*gra2*, RHΔ*gra2*, and RHΔ*gra6* parasites (38, 53, 54), which failed to establish typical elongated IVN nanotubular membrane structures in their PV, our data showed that RHΔ*gra12* parasites still established IVN nanotubular membranes within their PV and that GRA2 still partitioned normally into the IVN membrane and the PV soluble fractions. Previous results had shown that PruΔ*asp5* parasites, which are deficient in the Golgi complex-associated aspartyl protease 5 (ASP5) required for the processing and targeting of many secreted GRA proteins, do differentiate to establish GFP^+^ bradyzoites. However, their cyst wall development is severely impaired when differentiation is induced by *in vitro* alkaline switching (71). Our data showed that PruΔ*gra12* parasites exhibited a relative delay in the accumulation of the CST1 major cyst wall protein at the cyst periphery, but this defect was mild in comparison to the severe defects previously reported in PruΔ*asp5* cysts. Together, these findings suggested that the nanotubular IVN membrane structures present within the PV do not appear to be strictly necessary for the development of the cyst wall. These findings should be interpreted with caution, because (i) certain IVN membrane-associated GRA proteins, for example, GRA12, may participate in cyst wall development and (ii) these *in vitro* results may not truly reflect what happens *in vivo*, where the development of cysts during toxoplasmosis is triggered by host immune responses (77–79).

In mature cysts isolated from mice chronically infected with a type II strain, PVM-associated (GRA3 and GRA7) and IVN-associated (GRA2 and GRA6) GRA proteins were observed at the cyst periphery (67). Proteomic analysis of the isolated *in vitro* cyst wall has recently revealed that many GRA proteins localize at the cyst wall (68), including the GRA2, GRA3, GRA4, GRA5, GRA7, GRA8, GRA9, GRA12, and GRA14 proteins evaluated in this study. Our hypothesis is that IVN and PV membrane-associated GRA proteins could play a structural or organizational role during cyst development or in cyst maintenance. Deletion of the major cyst wall protein CST1 (8), as well as gene knockout of a nucleotide sugar transporter or the glycosylation pathway that glycosylates CST1 and other cyst wall proteins, increased cyst fragility and decreased cyst burdens *in vivo* (80, 81). We did not observe any clear increase in the frequency of broken cysts after mechanical disruption of the mouse brains infected with our PruΔ*gra* mutants. Our finding that PruΔ*gra12* cysts exhibited a reduced intensity of DBA fluorescence at the cyst periphery compared to the intensity at the cyst interior suggested that GRA12 could regulate delivery or retention of the major cyst wall protein CST1 during the development of the cyst wall.

Defects in cyst burdens were not associated with a decrease in acute virulence in PruΔ*gra9* parasites, trended toward an association with reduced virulence in PruΔ*gra6* parasites, and were associated with markedly reduced virulence in PruΔ*gra2*, PruΔ*gra4*, and PruΔ*gra12* parasites. While our data identified a defect in acute virulence in PruΔ*gra3* parasites, we did not identify any acute virulence defect in PruΔ*gra7*, PruΔ*gra8*, or PruΔ*gra14* parasites. Our findings are consistent with previous reports that identified acute virulence defects in type I RHΔ*gra2* (57) and type II PruΔ*gra3* (82) parasites. While GRA6 and GRA7 were previously linked with virulence mechanisms in type I strains (19, 56), these virulence phenotypes were absent in type II strains. GRA6 was previously identified as a type I strain-dependent virulence factor that activates NFAT4 (56). The IRG resistance mechanisms in virulent type I strains also involved the GRA7 protein that was previously shown to prepare IRGs, through turnover, for neutralization by the PVM-localized ROP5/ROP17/ROP18 protein complexes (19, 20). Our finding that virulence is not associated with GRA7 in type II strains suggested that either GRA7 is a strain type-dependent virulence factor or the virulence phenotype, as in type I strains (19), is not visible in this type II mutant when ROP5 and ROP18 virulence functions are expressed. In addition to the defects observed in cyst wall development and in IVN nanotubular morphology, PruΔ*asp5* parasites also exhibit reduced virulence in mice (71, 83). Since the IVN nanotubular membrane structures are present in RHΔ*gra4* parasites (38) and, as we show here, in both RHΔ*gra12* and PruΔ*gra12* parasites, virulence phenotypes do not appear to be specifically correlated with the presence or absence of distinct IVN nanotubular membrane structures.

The severity of the virulence defect observed in both type I and type II Δ*gra12* parasites mirrored the severity of the virulence defect previously observed in high-virulence type I RHΔ*rop18* (25) and low-virulence type II PruΔ*rop18* parasites (27). Notably, the loss of PV viability of type I RHΔ*gra12* parasites was cell type dependent, since it was observed specifically in IFN-γ-activated macrophages but not in IFN-γ-activated dendritic cells or MEFs. These data are consistent with previous findings that showed that macrophages harbor the most effective IFN-γ-activated mechanisms that disrupt the viability of the PV (84). The loss of viability of type II PruΔ*gra12* PVs observed in IFN-γ-activated MEFs is most likely explained by the inherently less active ROP5 and ROP18 mechanisms that resist host IFN-γ in low-virulence type II strains (27). In such low-virulence strains, GRA12 was crucial to maintain resistance to host IFN-γ, whereas the essentiality of GRA12 in high-virulence type I strains was visible only in IFN-γ-activated macrophages that launch the most destructive attacks on the PV.

Type I RHΔ*gra12* and type II PruΔ*gra12* parasites exhibited no defect in their ability to resist increased coating of the PVM with Irgb6 in IFN-γ-activated macrophages. ROP5 and ROP18 virulence factors directly resist host effector IRGs, including the pioneer Irgb6 (73), by neutralizing them and preventing an increase in their PVM coating (23–25, 27). ROP18 also resists the recruitment of GBP1 and ubiquitin to the PV (34, 35). In addition to ROP18, other PVM-localized proteins resist the recruitment of GBPs to the PV. Type II parasites that do not express the PVM-localized rhoptry pseudokinase ROP54 failed to resist host GBP2 coating of the PVM, and PruΔ*rop54* parasites were attenuated in their virulence (85). The targeting of the *Toxoplasma* PV by IRGs and GBPs is coordinated with the functions of several host autophagy proteins and, together, they lead ultimately to the destruction of the PV (86). Our results demonstrated that the loss of Δ*gra12* PV viability was not observed in *Irgm1*^−/−^/*Irgm3*^−/−^ macrophages that fail to express IRGM1 and IRGM3 functions. IRGM1 and IRGM3 are regulatory IRG proteins that are essential for the activation of host effector IRGs that can disrupt the PV (74, 75, 87). Regulatory IRGM proteins also regulate autophagic flux and influence the localization of GBP2 by modulating macroautophagy (88). In addition, loss of IRGM1 and IRGM3 expression induces a failure to target both IRGs and GBPs to the *Toxoplasma* PV (89). However, while regulatory IRGM1 and IRGM3 proteins have been shown to control the pathogen-killing mechanisms of IRG effectors (74, 75, 87), it is currently unknown whether regulatory IRGM proteins specifically regulate functions of GBP proteins that can restrict the PV. Therefore, additional studies are necessary to elucidate the specific roles of regulatory IRGM1 and IRGM3, effector IRGs, GBPs, and autophagic machinery in the restriction of PVs that lack expression of GRA12.

Interestingly, GRA12 was previously identified in proteomic analysis of ROP5 and ROP18 high-molecular-weight protein complexes but was not found associated with ROP17 protein complexes (GRA12 is shown in Table 1 of reference 24 as TGGT1_034740). In addition, BirA* proximity localization studies using a PVM-localized GRA17-BirA* fusion protein also identified GRA12 as one of the interacting partners of GRA17 (90). These proteomic studies suggested that GRA12 closely interacted with several protein complexes that localized at the PVM, which agrees with our observations that GRA12 sporadically colocalized with PVM-associated GRA5 and with previously reported data showing that GRA12 sporadically colocalized with GRA3 at the PVM (52). Taking into account that (i) GRA12 predominantly localized at the IVN rather than the PVM, (ii) GRA12 sporadically localized with PVM-localized proteins GRA3 (52) and GRA5, (iii) GRA12 was identified in PVM-localized protein complexes in association with GRA17 (90) or ROP5/ROP18 (24), (iv) within the PV, the IVN membranes form direct connections with the PVM (45), (v) Δ*gra12* PVs resisted early coating of the PV by host IRGs in IFN-γ-activated macrophages, (vi) Δ*gra12* PVs succumbed in an IRGM1/IRGM3- and IFN-γ-dependent fashion, and (vii) Δ*gra12* cysts accumulated less CST1 cyst wall protein at the cyst periphery relative to the amount in the cyst interior *in vitro*, we hypothesized that GRA12 is likely to be involved in the delivery or exchange of protein or lipid cargo between the IVN membranes and the PVM to support PVM resistance to host IFN-γ.

The PVM is a dynamic molecular interface separating the PV lumen from the host cell cytosol. The PVM is decorated with important protein complexes, such as the GRA17/GRA23 pore protein complexes (91), the ROP5/ROP18 protein complexes that allow resistance to IFN-γ-activated IRGs (25) and GBPs (34, 35), and the MYR1/MYR2/MYR3 protein complexes that translocate parasite GRA proteins across the PVM to modulate host gene transcription (92–94). GRA12 is mostly localized to the IVN and does not appear to be translocated past the PVM, suggesting it is unlikely that GRA12-mediated defense against IFN-γ is achieved through direct GRA12 protein interactions with host cell molecules. Since host cell IRGM1 and IRGM3 functions were necessary to make PVs that lack GRA12 expression nonviable, we can infer that GRA12 is required to support the functions of PVM-localized protein complexes in resisting host IFN-γ to maintain PV viability. Collectively, our results demonstrated the importance of many PV membrane GRA proteins in establishing chronic infection and revealed a virulence mechanism mediated by IVN membrane-associated GRA12 that underpins parasitophorous vacuole resistance to host IFN-γ.

## MATERIALS AND METHODS

### Parasite and host cell cultures.

Human foreskin fibroblasts (HFF) were amplified in Eagle’s modified essential medium (EMEM) complemented with 10% fetal bovine serum (FBS) (HyClone), 2 mM glutamine, 100 units/ml penicillin, and 100 μg/ml streptomycin. Confluent HFF monolayers were infected with T. gondii strains RH*Δku80* (72, 95) and Pru*Δku80* (66) and knockout or mutant strains derived from these parental strains in EMEM containing 1% FBS, 2 mM glutamine, 100 units/ml penicillin, and 100 μg/ml streptomycin, as previously described (96, 97). Mouse embryonic fibroblasts (MEFs) derived from C57BL/6 mice (ATCC) were cultured in Dulbecco’s modified Eagle’s medium (DMEM) supplemented with 15% FBS, 2 mM glutamine, 100 units/ml penicillin, and 100 μg/ml streptomycin. Bone marrow-derived dendritic cells (BMDC) and macrophages (BMDM) were isolated from the femur and tibia of C57BL/6 mice or from C57BL/6 mice that lack functional *Irgm1* and *Irgm3* genes (*Irgm1*^−/−^/*Irgm3*^−/−^ knockout mice [74]). BMDM were differentiated in DMEM supplemented with 10% FBS, 1× minimal essential medium with nonessential amino acids, 1 mM sodium pyruvate (Life Technologies), 100 units/ml penicillin, 100 μg/ml streptomycin, and 30% L929 culture supernatant, as previously described (98). BMDC were differentiated in Roswell Park Memorial Institute (RPMI) 1640 medium (Fisher Scientific) supplemented with 10% FBS, 100 units/ml penicillin, 100 μg/ml streptomycin, 50 μM 2-mercaptoethanol (Sigma), and 20 ng/ml murine granulocyte-macrophage colony–stimulating factor (GM-CSF) (Peprotech), as previously described (99). BMDC were harvested for experiments 9 days after differentiation, while BMDM were harvested after 5 days of differentiation.

### Mice.

Female 7- to 9-week-old C57BL/6 mice and IFN-γ^−/−^ C57BL/6 mice were obtained from Jackson Laboratories (Bar Harbor, ME). Female 7- to 9-week-old CD1 mice were obtained from Charles River Laboratories (Wilmington, MA).

### Acute virulence assay.

High-viability type I or type II tachyzoites were isolated from 3-day-infected HFF cultures as previously described (66, 100). Parasites were centrifuged at 900 × *g* for 7 min, washed, and counted in Dulbecco’s modified phosphate-buffered saline (DPBS). Parasite viability was confirmed in HFF plaque assays (101). Groups of 4 C57BL/6 mice (type II infection) were injected intraperitoneally (i.p.) with 2 × 10^5^, 2 × 10^6^, or 2 × 10^7^ tachyzoites, or groups of 4 CD1 mice (type I infection) were injected intraperitoneally with 2 × 10^2^ tachyzoites. In experiments using mice deficient in the production of IFN-γ (IFN-γ^−/−^ mice), groups of 4 mice were injected intraperitoneally with 2 × 10^2^ parasites. Mice were monitored for symptoms of infection, weight loss, and mortality for 30 days, and survival was evaluated using the Kaplan-Meier curve.

### Cyst burden assays.

High-viability type II tachyzoites were obtained as previously described (66, 100). Tachyzoites were centrifuged at 900 × *g* for 7 min, washed, and counted in DPBS. Groups of 4 mice were infected by intraperitoneal (i.p.) injection with 2 × 10^2^ parasites, and parasite viability was confirmed in a plaque assay. Mice were monitored for symptoms of infection, weight loss, and mortality for 21 days. The brains from mice infected with type II strains were harvested at 3 weeks postinfection and homogenized using a Dounce homogenizer in 2 ml of sterile DPBS. The cysts from a minimum of 10% of each brain were scored. Since Pru strain background cysts can vary in size (66, 102), they were scored with dark-field microscopy using an inverted phase-contrast fluorescence microscope (Olympus CKX41). The PruΔ*ku80* parent strain expresses GFP under the control of the bradyzoite stage-specific *LDH2* promoter (66). GFP^+^ cysts were scored using a total magnification power of ×150 because this magnification provided the highest sensitivity for the detection of GFP^+^ bradyzoites within latent cysts. GFP^+^ cysts were then observed in bright-field microscopy at ×300 total magnification to verify that these cysts possessed a thick translucent cyst wall that completely surrounded the GFP^+^ bradyzoites (66, 100).

### Deletion of *GRA* genes.

Targeted *GRA* gene of interest (GOI) deletions (Table S1) were developed using the Δ*ku80* knockout strain of the type II Prugniaud strain (PruΔ*ku80*) or the type I RH strain (RHΔ*ku80*), as previously described (see Table S1 in the supplemental material) (27, 66, 72). Briefly, *GRA* gene locus knockout-targeting plasmids were assembled in the yeast shuttle vectors pRS416 or pRS426 using yeast recombination cloning to fuse, in the following order, 3 distinct PCR products with 31- to 34-bp crossovers: a 5′ *GRA* GOI target-flanking sequence, the *HXGPRT* selectable marker, and a 3′ *GRA* GOI target-flanking sequence (Fig. S1A) (100). Knockout plasmids were engineered to delete at least 200 nucleotides of the 5′ untranslated region (UTR) and the complete coding sequence of the *GRA* GOI locus as defined in the ToxoDB.org database (103). All oligonucleotide primers used to construct the knockout-targeting plasmids and the ToxoDB nucleotide definitions of deleted *GRA* gene loci are listed in Table S2. The targeting plasmids were validated by DNA sequencing and were linearized at restriction sites inserted at the 5′ end of the 5′ target-flanking sequence (Fig. S1A). The linearized targeting plasmids were transfected by electroporation into tachyzoites of the PruΔ*ku80* strain. *GRA* GOI knockouts were selected with 50 μg/ml mycophenolic acid and 50 μg/ml xanthine. Drug-selected parasite mutants were cloned by limiting dilution 30 days after transfection. *GRA* GOI knockouts were validated by genotype analysis using the PCR strategy shown in Fig. S1A, as follows: (i) PCR 1 verified targeted deletion of the coding region of the targeted gene (DF and DR primers), (ii) PCR 2 verified the correct targeting of the 5′-end integration (CXF and 5′DHFRCXR primers), and (iii) PCR 3 verified the correct targeting of the 3′-end integration (3′DHFRCXF and CXR primers). All the knockout validation primers are shown in Table S2.

10.1128/mBio.00589-19.7 TABLE S2 Primers used to construct and validate GRA knockouts. Download Table S2, DOCX file, 0.04 MB. Copyright © 2019 Fox et al. 2019 Fox et al. This content is distributed under the terms of the Creative Commons Attribution 4.0 International license.

### Complementation of Δ*gra12* parasite strains.

Complementation plasmids were designed to complement the PruΔ*gra12 and* RHΔ*gra12* strains through targeted chromosomal integration and expression of wild-type or mutant gene alleles at the uracil phosphoribosyltransferase (*UPRT*) chromosomal locus (TGME49_312480), as previously described (Fig. S1B) (27, 66). Complementation plasmids were developed in the pRS416 or pRS426 yeast shuttle vectors using yeast recombination to fuse, in this order, a 5′ *UPRT* target-flanking sequence, the complementing gene of interest with its native 5′ UTR, and the 3′ *UPRT* target-flanking sequence (Fig. S1B). Oligonucleotide DNA primers (Table S3) were used to generate the complementing genes, synthesized as one PCR product. Following plasmid assembly by yeast recombinational cloning, the targeting plasmids were validated by DNA sequencing. Prior to transfection, plasmids were linearized at the unique restriction site PmeI. Following transfection, parasites were cultured for 2 days in normal infection medium, and the cultures were then switched to selection medium containing 2 μM 5-fluorodeoxyuridine (FUDR). Thirty days after transfection, parasites were subcloned by limiting dilution. Accurate targeting of complementing genes into the *UPRT* locus was validated by genotype analysis using 4 PCRs (strategy shown in Fig. S1B), of which (i) PCR 4 verified the deletion of the *UPRT* coding region, (ii) PCR 5 verified correctly targeted 5′-end integration, and (iii) PCR 6 verified correctly targeted 3′-end integration of the complementing transgene at the *UPRT* locus. All the oligonucleotide DNA validation primers are shown in Table S3.

10.1128/mBio.00589-19.8 TABLE S3 Primers used to construct plasmids for complementation and validation of GRA mutant strains. Download Table S3, DOCX file, 0.03 MB. Copyright © 2019 Fox et al. 2019 Fox et al. This content is distributed under the terms of the Creative Commons Attribution 4.0 International license.

### Validation of knockouts by indirect IFA and immunoblotting.

Parasites were isolated from freshly lysed cultures and suspended in Laemmli buffer. Proteins were separated by 13% SDS-PAGE (nonreduced conditions), transferred to nitrocellulose membranes, and detected using rat anti-GRA12 antibodies (1:400) (52). Proteins were detected with horseradish peroxidase (HRP)-conjugated secondary antibodies (1:20,000; Jackson Immunoresearch Laboratories), and the peroxidase activity was visualized by chemiluminescence using the SuperSignal enhanced chemiluminescence (ECL) system (Pierce Chemical). For immunofluorescence assay (IFA), confluent HFF cells were grown on glass coverslips and infected for 24 h. Infected cells were fixed in 5% paraformaldehyde for 20 min, permeabilized for 10 min in 0.002% saponin, and blocked in 5% goat serum and 5% FBS in PBS. Cells were incubated with primary antibodies for 1 h in DPBS containing 1% FBS and 0.002% saponin. The primary antibodies used in IFA validation of GRA knockouts included the following mouse monoclonal antibodies (MAbs): anti-GRA2 MAb TG17.179 (1:500) (47), anti-GRA3 MAb T6.2H11 (1:500) (104), rabbit anti-GRA4 MAb (1:500) (49), rabbit anti-GRA6 MAb (1:500) (49), anti-GRA7 MAb BATO 214 (1:500) (105), anti-GRA8 MAb 3.2 (1:500) (106), rabbit anti-GRA9 MAb (1:250) (51), rat anti-GRA12 MAb (1:400) (52), mouse polyclonal anti-GRA14 MAb (1:500) (40), rabbit antiactin MAb (1:500) (107), or anti-SAG1 MAb TG054 (1:500) (108). (Antibodies were purchased from the Biotem company, Apprieu, France, or kindly provided by L. D. Sibley, Washington University School of Medicine, Saint-Louis, MO, D. Jacobs, Innogenetics-Fujirebio Europe N.V., Ghent, Belgium, G.E. Ward, University of Vermont College of Medicine, Burlington, VT, W. Daübener, Heinrich Heine Universität, Düsseldorf, Germany, M. Lebrun, Montpellier, France, and Peter Bradley, University of California, Los Angeles, CA). Infected cells were washed and incubated for 1 h with goat anti-mouse IgG(H+L)-Alexa Fluor 488 (1:500) (Jackson), goat anti-mouse IgG(H+L)-Alexa Fluor 594 (1:500) (Jackson), goat anti-rat IgG(H+L)-Alexa Fluor 488 (1:500) (Jackson), or goat anti-rabbit IgG(H+L)-Alexa Fluor 488 (1:500) (Jackson) secondary antibody. Coverslips were then incubated in 5 μg/ml Hoechst 33342 to stain nuclei, mounted in Mowiol, and observed using an Axioplan II microscope (Zeiss) equipped with a 100× objective. Images were acquired with a black-and-white camera (Zeiss) and AxioVision version 4.7.1.

### Intracellular replication rate assay.

The parasite growth rate was determined using a previously described method that scores the number of parasites per vacuole (66). Briefly, triplicate monolayers of HFF cells were infected at a multiplicity of infection (MOI) of ∼0.2 and parasites were let to invade cells for 1 h. Monolayers were then washed 3 times in PBS to remove extracellular parasites. The number of tachyzoites per vacuole was scored in at least 50 PVs randomly encountered on the coverslip at 45 h postinfection for type II parasite strains or at 30 h postinfection for type I parasite strains.

### PV viability assay.

BMDC or BMDM were collected and seeded overnight into 6-well plates at a density of 5 × 10^6^ cells per well. Triplicate sets of BMDC or BMDM cultures were incubated for 4 to 6 h in complete medium or in activation medium containing murine IFN-γ (100 U/ml; Peprotech) and TNF-α (10 U/ml; Peprotech). Cells were infected with 100 parasites per well, and cultures were left undisturbed for 6 days at 37°C. The medium was aspirated from each well, and the BMDC and BMDM monolayers were fixed in 50% methanol and 10% acetic acid and stained with 0.25% Coomassie brilliant blue to visualize plaques. The number of plaques in each well was scored. MEFs were seeded into 24-well plates at a density of 1 × 10^5^ cells per well and reached confluence in ∼24 h. MEF control monolayers were incubated without IFN-γ or MEFs were stimulated in activation medium containing 200 U/ml IFN-γ (Peprotech) 24 h prior to parasite infection. Triplicate wells were infected with 200 or 1,000 parasites, and plaques were let to develop for 5 to 7 days. The total number of PFU per well was counted microscopically, scoring all the PFU in each well. The percentage of PV viability was calculated to represent the number of PFU in IFN-γ-activated cells divided by the number of PFU scored in control not-activated cells.

### IRG coating assay.

BMDM and BMDC were harvested, seeded onto circular glass coverslips (Electron Microscopy Sciences), and incubated overnight. BMDM and BMDC were then activated with 100 U/ml IFN-γ and 10 U/ml TNF-α for 6 h and infected for 45 min with parasites at an MOI of 4. The coverslips were washed in DPBS, and cells were fixed with 4% paraformaldehyde (Electron Microscopy Sciences), permeabilized with 0.1% saponin (Sigma), and blocked in 10% FBS. For visualization, cultures were incubated with mouse anti-GRA5 antibody (Ab) (TG 17.113 at 1:2,000; Biotem) and rabbit anti-Irgb6 Ab (1:1,000) (74), washed, and incubated with anti-mouse IgG(H+L) coupled to Alexa Fluor 568 and anti-rabbit IgG(H+L) coupled to Alexa Fluor 488 as secondary antibodies (Invitrogen). Coverslips were mounted in ProLong gold with DAPI (4′,6-diamidino-2-phenylindole; Invitrogen) and imaged at ×63 magnification with a Nikon A1R SI confocal microscope (Nikon, Inc.). All images were processed using the FIJI program (109). A minimum of 500 PVs of each strain was scored for quantification of Irgb6 coating of the PVM, as previously described (27).

### IFA colocalization assays.

HFF cells were cultured on circular glass coverslips (Electron Microscopy Sciences) and infected with parasites for 24 h. For visualization of dense granule proteins in the PV, cultures were fixed in 4% paraformaldehyde and permeabilized for 10 min with 0.01% saponin (Sigma). All samples were blocked for 1 h with 10% FBS and incubated for 1 h with a 1:500 dilution of primary rabbit anti-HA tag MAb (Cell Signaling) and a 1:1,000 dilution of primary mouse anti-GRA2 MAb or anti-GRA5 MAb (Biotem, France). Preparations were washed 3 times with DPBS supplemented with Ca^2+^ and Mg^2+^ and incubated for 1 h at room temperature with a 1:1,000 dilution of goat anti-rabbit and goat anti-mouse IgG secondary antibodies conjugated to Alexa Fluor 488 and Alexa Fluor 594, respectively. Samples were mounted in SlowFade gold antifade with DAPI (Life Technologies) and imaged with a Nikon A1R SI confocal microscope (Nikon, Inc.). PVs were located using differential interference contrast microscopy (DIC). Confocal images were processed using the FIJI program (109).

### *In vitro* cyst differentiation assay.

Tachyzoite-to-bradyzoite differentiation using *in vitro* alkaline switch was performed as described by Knoll and colleagues (110). The differentiation medium consists of RPMI 1640 without bicarbonate and supplemented with 2.05 mM l-glutamine (HyClone), 20 mM HEPES free acid (IBI Scientific), 1% Glutamine XL (a stable form of glutamine; VWR), 1% FBS, and 1% penicillin-streptomycin. The differentiation medium pH was adjusted to 8.1 with sodium hydroxide. Monolayers of HFF cells were cultured on circular glass coverslips until confluent and infected with type II parasite strains at an MOI of ∼0.5. Infected cells were washed once 3 h later with DPBS supplemented with Ca^2+^ and Mg^2+^, and cultures were returned to high-pH differentiation medium for 3 days at 37°C in ambient air to differentiate cysts. Infected cells were fixed in 4% paraformaldehyde, and the excess of paraformaldehyde was quenched with 0.1 M glycine. Infected cells were simultaneously permeabilized and blocked for 30 min at room temperature in 3% FBS–0.2% Triton X-100 and incubated for 1 h at room temperature with a 1:250 dilution of rhodamine-labeled Dolichos biflorus agglutinin (DBA) (Vector Laboratories). Preparations were washed three times with DPBS, mounted in SlowFade gold antifade with DAPI (Life Technologies), and imaged with a Nikon A1R SI confocal microscope (Nikon, Inc.).

### *In vitro* cyst DBA fluorescence intensity quantification assay.

Cysts (≥10) matured *in vitro* for 3 days were located by DIC microscopy. Images were imported into FIJI to evaluate the fluorescence intensity of rhodamine-labeled DBA in cysts. All measurements were taken at the zoom magnification of 300% to allow more accurate manual drawing separation of the cyst periphery (cyst wall) from the cyst interior (that does not include the cyst wall). First, we defined the region of interest by using the freehand selection tool to draw a circle around the entire cyst and to capture the total DBA signal. To measure fluorescence, both the DBA color channel and the DIC channel (to define the area being measured) were selected. Under the set measurements tab, the following parameters were selected: area, integrated density, and mean gray value. The region of interest was analyzed by selecting measure, and measurements for area, integrated density, and mean gray value were recorded in a table. This was repeated, independently, 3 times for each cyst. In a second step using the freehand selection tool, the region inside the cyst was defined by drawing, beneath the cyst wall, a circle that excluded the DBA fluorescence of the cyst wall. Similarly, this was repeated, independently, 3 times for each cyst. In a third step, to measure the background fluorescence, a circle was drawn close to the cyst and measured, and this was repeated 3 independent times, each time rotating the cyst aspect before each measurement. The mean fluorescence of background readings was obtained by calculating the average of the three mean gray value numbers collected. In the fourth step, the corrected total cyst fluorescence (CTCF) was calculated for each cyst region measurement by using the equation CTCF = integrated density − (area of selected cyst × mean fluorescence of background readings). To capture an estimate of the cyst wall, the CTCF of the cyst periphery region was calculated by subtracting the average CTCF of the cyst interior region from the average CTCF of the entire cyst. Finally, for each cyst, the cyst periphery CTCF was divided by the cyst interior CTCF to quantify the DBA fluorescence intensity at the cyst periphery relative to that of the cyst interior. All the ratios (10 PruΔ*ku80* cysts and 12 PruΔ*gra12* cysts) were graphed using the Prism program, and significance was evaluated using the unpaired *t* test.

### TEM.

HFF monolayers were grown to confluence on Permanox slides in DMEM supplemented with 10% FBS and 1% sodium pyruvate (D10 medium). Cells were infected for 24 h in D10 medium, rinsed with PBS, fixed for 2 h with glutaraldehyde diluted in 0.2 M NaPO_4_, pH 7.4, and processed for transmission electron microscopy (TEM) as previously described (54). The infected cells were flat embedded and sectioned *en face* to enhance the preservation of the IVN structures.

### PV fractionation of infected host cells.

PV membranes were physically separated from PV soluble molecules in infected HFF cells using a previously described method (45). Briefly, HFF monolayers in 150-cm^2^ flasks were infected for 24 h at an MOI of 3. Infected HFFs were gently washed once with DPBS supplemented with Ca^2+^ and Mg^2+^ to remove residual cell culture medium. Infected cell monolayers were dislodged in cold DPBS supplemented with Ca^2+^ and Mg^2+^ in the presence of a cocktail of protease inhibitors (Roche). Cells were recovered by low-speed centrifugation, and infected cells were mechanically disrupted by syringing through a 27-gauge needle to break host cells and PVs and to release still-intact parasites. Parasites and large host cell debris were pelleted by low-speed centrifugation at 2,500 × *g* for 10 min. The supernatants containing the soluble components of the infected cells and the PV membranes were then fractionated by 1 h of ultracentrifugation at 100,000 × *g* into a soluble high-speed supernatant (HSS) and a high-speed pellet (HSP) containing the PV membranes. Equal fractions of the HSP and the HSS were boiled in Tris-glycine SDS sample buffer (Novex) containing 2-β-mercaptoethanol and separated on 10% Tris-glycine WedgeWell gels (Novex). Gels included lanes with a prestained protein ladder (PageRuler) and a biotinylated protein ladder (Cell Signaling) and were later visualized using antibiotin antibodies (1:1,000) conjugated to horseradish peroxidase. The proteins were transferred to nitrocellulose membranes using a semidry Trans-Blot turbo transfer system (Bio-Rad). Membranes were blocked for 1 h with 5% (wt/vol) nonfat dry milk in 1× Tris-buffered saline (10 mM Tris HCl and 150 mM NaCl at pH 7.4) containing 0.1% Tween 20 (TBS-T buffer). Membranes were incubated overnight at 4°C with primary rabbit anti-HA antibodies (1:1,000) (Cell signaling) or anti-GRA2 MAb (1:1,000) (Biotem). After 3 washes in 1× TBS-T buffer for 5 min, membranes were incubated for 1 h with secondary goat anti-rabbit IgG (1:2,000) or goat anti-mouse IgG (1:2,000) conjugated to horseradish peroxidase. After 3 washes in 1× TBS-T buffer for 5 min each, signals were detected with LumiGlo chemiluminescence reagent (Cell signaling) and exposed to X-ray film.

### Statistical analyses.

Statistical analyses were performed using the GraphPad Prism 6.0 software. Statistical data for bar graphs were calculated using 2-tailed unpaired Student’s *t* tests, which were conducted using the assumption of equal variance. The standard errors of the means (SEM) from independent samples assayed within the represented experiments are reported. Survival experiments were analyzed using the log rank Mantel-Cox test.

### Ethics statement.

All animal experiments were conducted in accordance with the recommendations in the *Guide for the Care and Use of Laboratory Animals* (111) and the Association for the Assessment and Accreditation of Laboratory Animal Care (AAALAC) guidelines. Animals were housed in an AAALAC-certified facility, and animal protocols were approved by the Dartmouth College Committee on the Use and Care of Animals.
