# Supplementary figures and images for: Toxoplasma gondii Parasitophorous Vacuole Membrane-Associated Dense Granule Proteins Orchestrate Chronic Infection and GRA12 Underpins Resistance to Host Gamma Interferon
Source: mBio. 2019 Jul 2;10(4):e00589-19. doi: 10.1128/mBio.00589-19 (PMC6606796; doi:10.1128/mBio.00589-19)

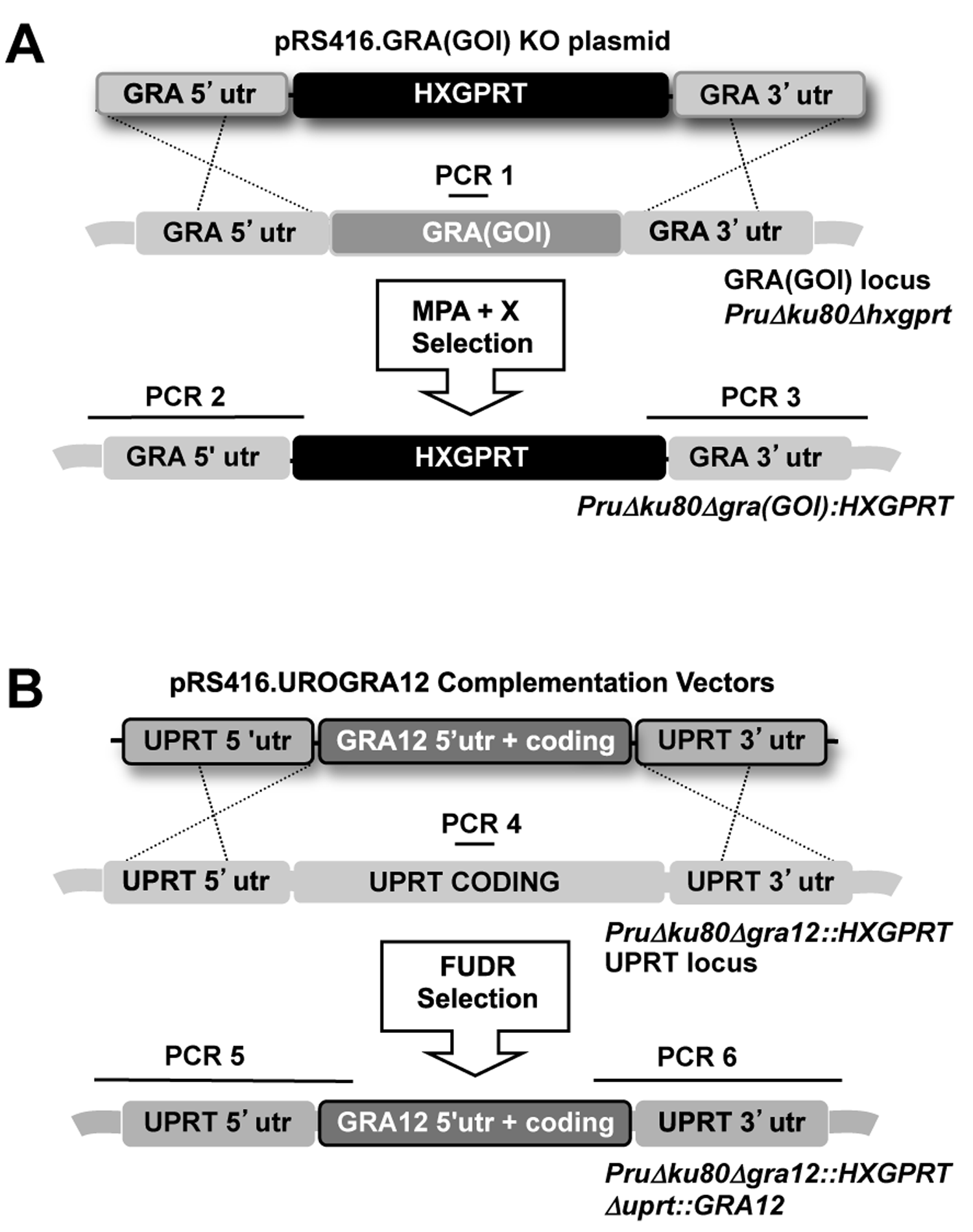

Supplement: FIG S1 [file mBio.00589-19-sf001.tif]

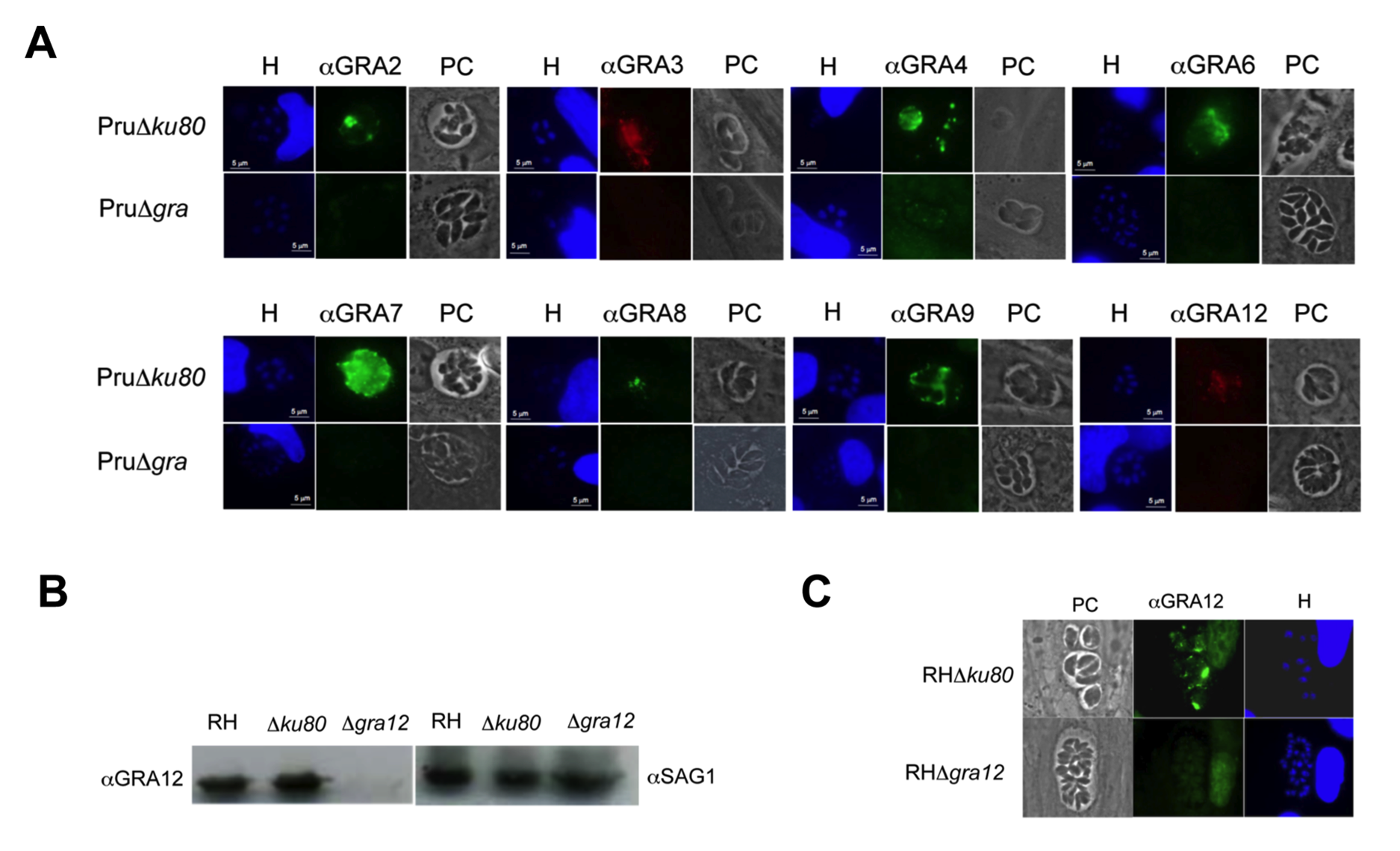

Supplement: FIG S2 [file mBio.00589-19-sf002.tif]

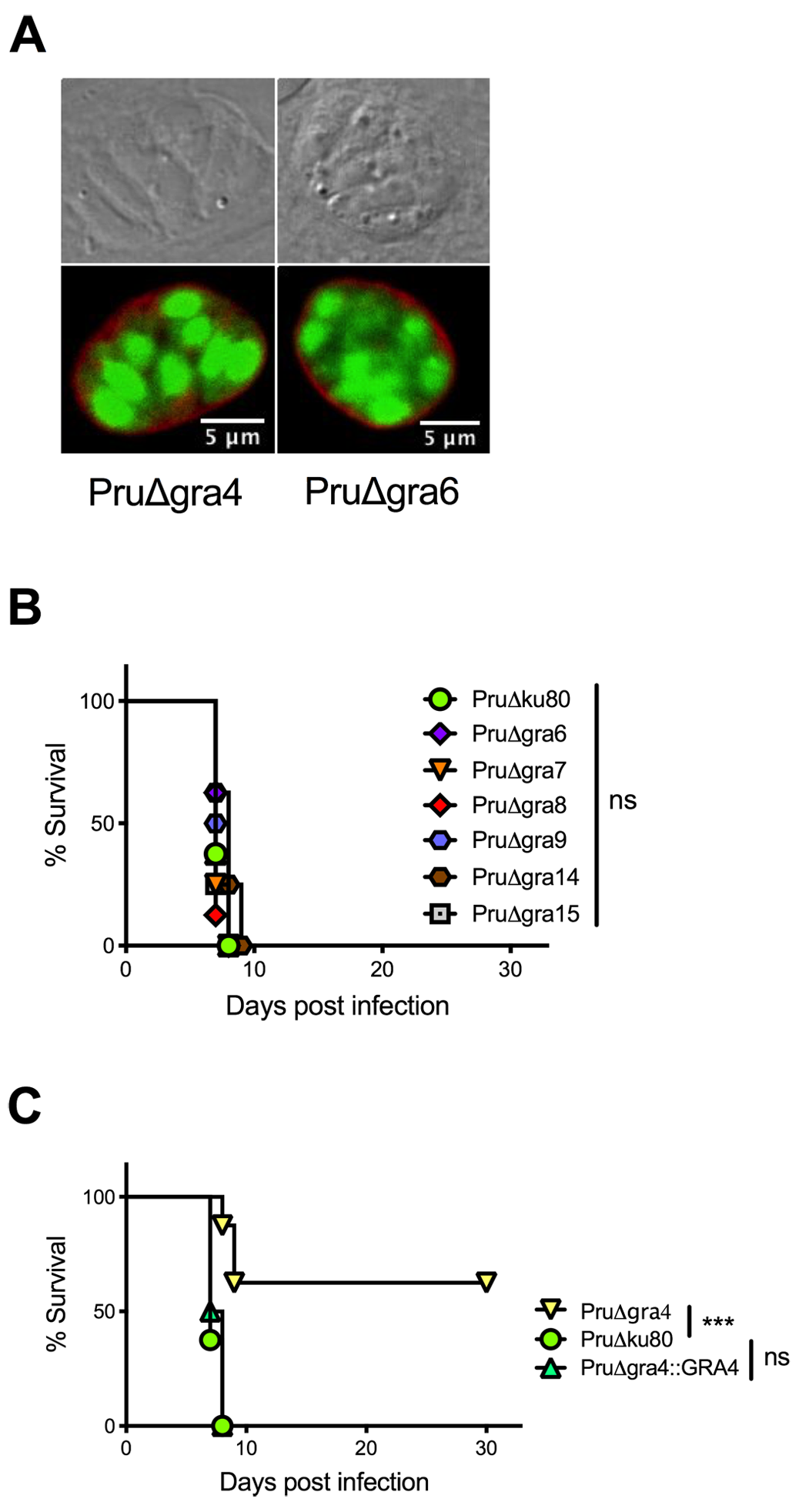

Supplement: FIG S3 [file mBio.00589-19-sf003.tif]

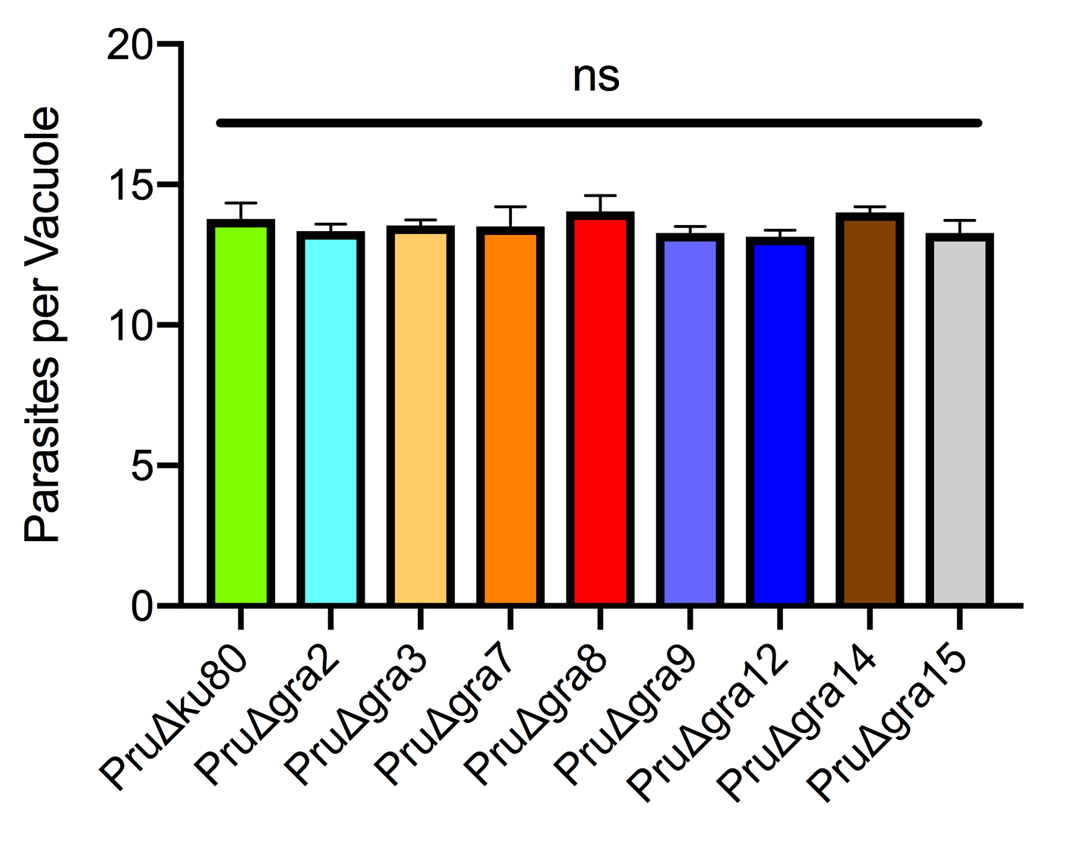

Supplement: FIG S4 [file mBio.00589-19-sf004.tif]
