# Supplementary material for: Toxoplasma gondii Parasitophorous Vacuole Membrane-Associated Dense Granule Proteins Orchestrate Chronic Infection and GRA12 Underpins Resistance to Host Gamma Interferon
Source: mBio. 2019 Jul 2;10(4):e00589-19. doi: 10.1128/mBio.00589-19 (PMC6606796; doi:10.1128/mBio.00589-19)
Supplement: TABLE S1 [file mBio.00589-19-st001.doc]

**Table S1. S**trains used in this study

| **Strain Designation** | **Complete Genotype** | **Source** |
| --- | --- | --- |
| PruΔku80 | PruΔ*ku80*Δ*hxgprt* | (1) |
| PruΔgra2 | PruΔ*ku80*Δ*gra2*::*HXGPRT* | (This study) |
| PruΔgra3 | PruΔ*ku80*Δ*gra3*::*HXGPRT* | (This study) |
| PruΔgra4 | PruΔ*ku80*Δ*gra4*::*HXGPRT* | (This study) |
| PruΔgra6 | PruΔ*ku80*Δ*gra6*::*HXGPRT* | (1) |
| PruΔgra4Δgra6 | PruΔ*ku80*Δ*gra6*Δ*gra4*::*HXGPRT* | (1) |
| PruΔgra4/GRA4 | PruΔ*ku80*Δ*gra4*::*HXGPRT*Δ*uprt::GRA4* | (1) |
| PruΔgra7 | PruΔ*ku80*Δ*gra7*::*HXGPRT* | (This study) |
| PruΔgra8 | PruΔ*ku80*Δ*gra8*::*HXGPRT* | (This study) |
| PruΔgra9 | PruΔ*ku80*Δ*gra9*::*HXGPRT* | (This study) |
| PruΔgra12 | PruΔ*ku80*Δ*gra12*::*HXGPRT* | (This study) |
| PruΔgra12/GRA12_II-HA_ | PruΔ*ku80*Δ*gra12::HXGPRTΔuprt*::*GRA12_II-HA_* | (This study) |
| PruΔgra12/GRA12_II-HA_ΔPLD | PruΔ*ku80*Δ*gra12::HXGPRT*Δ*uprt*::*GRA12_II-HA_*Δ*PLD* | (This study) |
| PruΔgra12/GRA12_I-HA_ | PruΔ*ku80*Δ*gra12::HXGPRT*Δ*uprt*::*GRA12_I-HA_* | (This study) |
| PruΔgra14 | PruΔ*ku80*Δ*gra14*::*HXGPRT* | (This study) |
| PruΔgra15 | PruΔ*ku80*Δ*gra15*::*HXGPRT* | (This study) |
| RHΔku80 | RHΔ*ku80*Δ*hxgprt* | (2) |
| RHΔgra12 | RHΔ*ku80*Δ*gra12*::*HXGPRT* | (This study) |
| RHΔgra12/GRA12_I-HA_ | RHΔ*ku80*Δ*gra12::HXGPRT*Δ*uprt*::*GRA12_I-HA_* | (This study) |
|  |  |  |

**Supplemental References**

1. Fox BA, Falla A, Rommereim LM, Tomita T, Gigley JP, Mercier C, Cesbron-Delauw MF, Weiss LM, Bzik DJ. 2011. Type II Toxoplasma gondii KU80 knockout strains enable functional analysis of genes required for cyst development and latent infection. Eukaryot Cell 10:1193-206.

2. Fox BA, Ristuccia JG, Gigley JP, Bzik DJ. 2009. Efficient gene replacements in Toxoplasma gondii strains deficient for nonhomologous end joining. Eukaryot Cell 8:520-9.
