# Supplementary material for: Toxoplasma gondii Parasitophorous Vacuole Membrane-Associated Dense Granule Proteins Orchestrate Chronic Infection and GRA12 Underpins Resistance to Host Gamma Interferon
Source: mBio. 2019 Jul 2;10(4):e00589-19. doi: 10.1128/mBio.00589-19 (PMC6606796; doi:10.1128/mBio.00589-19)
Supplement: FIG S5 [file mBio.00589-19-sf005.docx]

**Amino acid similarity and identity between Type I, II, and III GRA12 protein sequences**

GT1.Type_I 1 MRAIMASTQQFLRPVGSRPLAGISVVVVVALAIALGTAADVGRHVGGFSGPFPMTLRSGQ 60

ME49.Type_II 1 MRAIVASTQQFLRPVGSRPLAGISIVVVVALAIALGTAADVGRHVGGFSGPFPMTLRSGQ 60

VEG.Type_III 1 MRAIMASTQQFLRPVGSRPLAGISVVVVVALAIALGTAADVGRHVGGFSGPFPMTLRSGQ 60

****.*******************.***********************************

GT1.Type_I 61 WRLDHGACFVGKAKNLVVDPLPRVSPQGPQPLDVTTTGSALCWWLDSMYAAHMSLKAAWE 120

ME49.Type_II 61 WRLDHGACFVGKAKNLVVDPLPRVSPQGPQPLDVTTTGSALCWWLDSMYAAHMSLKAAWE 120

VEG.Type_III 61 WRLDHGACFVGKAKNLVVDPLPRVSPQGPQPLDVTTTGSALCWWLDSMYAAHMSLKAAWE 120

************************************************************

GT1.Type_I 121 KRHQEAKNRTSWLNLWRRFANWWASFPEFQIDVSVLYLDLWNDDFYGHPLPWFSAEFSYT 180

ME49.Type_II 121 RRHQEAKNRTSWLNLWRRFANWWASFPEFQIDVSVLYLDLWNDDFYGHPLPWFSAEFSYT 180

VEG.Type_III 121 KRHQEAKNRTSWLNLWRRFNNWWASFPEFQIDVSVLYLDLWNDDFYGHPLPWFSAEFSYT 180

.****************** ****************************************

GT1.Type_I 181 PPSGRYAYNLFDKLQSHFASAPGTAVQEVFLLLAPAPTFNQPVEKRSSIVARAATVAAGN 240

ME49.Type_II 181 PPSGRYAYNLFDKLQSHFASAPGTAVQEVFLLLAPAPTFNQPVEKRSSIVARAATVAAGN 240

VEG.Type_III 181 PPSGRYAYNLFDKLQSHFASAPGTAVQEVFLLLAPAPTFNQPVEKRSSIVARAATVAAGN 240

************************************************************

GT1.Type_I 241 ELFKEALGHQRVDEVLSMVPADPFRLMLSTSAFSFQAKIGDFWERGLDCMLGSRLNLRWD 300

ME49.Type_II 241 ELFKEALGHQRVDEVLSMVPADPFRLMLSTSAFSFQAKIGDFWERGLDCMLGSRLNLRWD 300

VEG.Type_III 241 ELFKEALGHQRVDEVLSMVPADPFRLMLSTSAFSFQAKIGDFWERGLDCMLGSRLNLRWD 300

************************************************************

GT1.Type_I 301 QVGTSVCRYMTAKASETGSGLAASFLNTVEVRVTGMDFFNHAAPVFKTEFIEGIITKRAT 360

ME49.Type_II 301 QVGTSVCRYMTAKASETGSGLAASFLNTVEVRVTGMDFFNHAAPVFKTEFIEGIITKRAT 360

VEG.Type_III 301 QVGTSVCRYMTAKASETGSGLAASFLNTVEVRVTGMDFFNHAAPVFKTEFIEGIITKRAT 360

************************************************************

GT1.Type_I 361 YIPVSMYLSTDPTLTHEYEAAKTVKRAVQAGRVGAALARGLVNFARATNQKADESHEGQT 420

ME49.Type_II 361 YIPVSMYLSTDPTLTHEYEAAKTVKRAVQAGRVGAALARGLVNFARATNQKADESHEGQT 420

VEG.Type_III 361 YIPVSMYLSTDPTLTHEYEAAKTVKRAVQAGRVGAALARGLVNFARATNQKADESHEGQT 420

************************************************************

GT1.Type_I 421 KTPTSGVRGSAGSKHN 436

ME49.Type_II 421 KTPTSGVRGSAGSKHN 436

VEG.Type_III 421 KTPTSGVRGSAGSKHN 436

****************

* = identical amino acid residue

. = similar amino acid residue

**ToxoDB: Gene annotations for GRA12:**

GT1 (Type I) GRA12 = TGGT1_288650

ME49 (type II) GRA12 = TGME49_288650

VEG (Type III) GRA12 = TGVEG_288650

**Amino acid similarity and identity between different strain types of GRA12:**

Type I and Type II GRA12 exhibit 100% similarity and 99.3% identity

Type I and Type III GRA12 exhibit 99.8% similarity and 99.8% identity

Typ II and Type III GRA12 exhibit 99.8% similarity and 99.1% identity
