# Supplementary material for: Toxoplasma gondii Parasitophorous Vacuole Membrane-Associated Dense Granule Proteins Orchestrate Chronic Infection and GRA12 Underpins Resistance to Host Gamma Interferon
Source: mBio. 2019 Jul 2;10(4):e00589-19. doi: 10.1128/mBio.00589-19 (PMC6606796; doi:10.1128/mBio.00589-19)
Supplement: TABLE S2 [file mBio.00589-19-st002.docx]

**Table S2. Primers used to construct and validate GRA knockouts.**

**Primer Sequence Primer Use Construct Corresponding locus KO**

**PMiniHXF** GATAAGCTTGATCAGCACGAAACCTTG HXGPRT cassette FP HXGPRT mini cassette NA

**PMiniHXR** CCGCTCTAGAACTAGTGGATCCC HXGPRT cassette RP

**GRA2PF1** *TTGGGTAACGCCAGGGTTTTCCCAGTCACGACG*GTTTAAAC**CTATGGAACAAGCCGTGTGTCGATAG** Pru Gra2 KO 5’ FP pRS416.pGRA2P TGME49_227620 chrX 870,704 to 871,574 (-)

**GRA2PR1** *GCGGGTTTGAATGCAAGGTTTCGTGCTGATCAA*ACTAGT**TCGCACTTAGCCTAAGGGACGAC** Pru Gra2 KO 5’ RP

**GRA2PF2** *TTCTGGCAGGCTACAGTGACACCGCGGTGGAGG*ACTAGT**TGGAGAGTGACAGAAGCACTACGAC** Pru Gra2 KO 3’ FP

**GRA2PR2** *GTGAGCGGATAACAATTTCACACAGGAAACAGC*GCGGCCGC**CGCCCTCGTGATTCCTCCAAG** Pru Gra2 KO 3’ RP

**GRA3PF1** *TTGGGTAACGCCAGGGTTTTCCCAGTCACGACG*GTTTAAAC**CATAACAACCCAGGTCTCGCGTC** Pru Gra3 KO 5’ FP pRS416.pGRA3P TGME49­_227280 chrX 1,049,334 to 1,050,088 (-)

**GRA3PR1** *GCGGGTTTGAATGCAAGGTTTCGTGCTGATCAA*ACTAGT**GTGCAGGTGTCCCACTGTCG** Pru Gra3 KO 5’ RP

**GRA3PF2** *TTCTGGCAGGCTACAGTGACACCGCGGTGGAGG*ACTAGT**GAGGCAACCCTTCATGAGTTCGG** Pru Gra3 KO 3’ FP

**GRA3PR2**  *GTGAGCGGATAACAATTTCACACAGGAAACAGC*CTCGAG**CGAGCAGCGATGTAGTTCACGATC** Pru Gra3 KO 3’ RP

**GRA7PF1** *TTGGGTAACGCCAGGGTTTTCCCAGTCACGACG*GTTTAAAC**CCCTGTGAGGTTCGTGCATCC** Pru Gra7 KO 5’ FP pRS416.pGRA7P TGME49_203310 chrVIIa 2,604,157 to 2,606,457 (+)

**GRA7PR1** *GCGGGTTTGAATGCAAGGTTTCGTGCTGATCAA*ACTAGT**ACGAAATCAGGGATCTCGCGC** Pru Gra7 KO 5’ RP

**GRA7PF2** *TTCTGGCAGGCTACAGTGACACCGCGGTGGAGG*ACTAGT**CAGAACTGTTCCTCGCGCTCG** Pru Gra7 KO 3’ FP

**GRA7PR2** *GTGAGCGGATAACAATTTCACACAGGAAACAGC*GCGGCCGC**GGCACCATATGGAAGGACCGTTG** Pru Gra7 KO 3’ RP

**GRA8F1** *TTGGGTAACGCCAGGGTTTTCCCAGTCACGACG*GTTTAAAC**GACATGCCGCCGGATCAACG** Pru Gra8 KO 5’ FP pRS416.pGRA8P TGME49_254720 chrIII 1,904,146 to 1,905,599 (+)

**GRA8R1** *GCGGGTTTGAATGCAAGGTTTCGTGCTGATCAA*ACTAGT**GGCTGGCAAGTTGGTTCGAACC** Pru Gra8 KO 5’ RP

**GRA8F2** *TTCTGGCAGGCTACAGTGACACCGCGGTGGAGG*ACTAGT**GTGAGGAGCGTCGATTGTGTCG** Pru Gra8 KO 3’ FP

**GRA8R2** *GTGAGCGGATAACAATTTCACACAGGAAACAGC*GCGGCCGC**CGCAGCGAATTGGGGCACAG** Pru Gra8 KO 3’ RP

**GRA9F1** *TTGGGTAACGCCAGGGTTTTCCCAGTCACGACG*GTTTAAAC**TCCGTATCACCCCTTCTTGGCC** Pru Gra9 KO 5’ FP pRS416.pGRA9P TGME49_251540 chrXII 5,450,182 to 5,453,129 (+)

**GRA9R1** *GCGGGTTTGAATGCAAGGTTTCGTGCTGATCAA*ACTAGT**GCAGAAGTGTGCGTCCGGTATC** Pru Gra9 KO 5’ RP

**GRA9F2** *TTCTGGCAGGCTACAGTGACACCGCGGTGGAGG*ACTAGT**CTGTTCGCTGATGTGAGTACGCC** Pru Gra9 KO 3’ FP

**GRA9R2** *GTGAGCGGATAACAATTTCACACAGGAAACAGC*GCGGCCGC**ACCTCGGGCAAGGAGAGTGG** Pru Gra9 KO 3’ RP

**GRA10PF1** *TTGGGTAACGCCAGGGTTTTCCCAGTCACGACG*GTTTAAAC**GGAACTCTGCAGGGAATGAGCG** Pru Gra10 KO 5’ FP pRS416.pGRA10P TGME49_268900 chrVIII 6,216,171 to 6,220,719 (-)

**GRA10PR1** *GCGGGTTTGAATGCAAGGTTTCGTGCTGATCAA*TCTAGA**TCCTGTGAAGAGGCACTATCGACC** Pru Gra10 KO 5’ RP

**GRA10PF2** *TTCTGGCAGGCTACAGTGACACCGCGGTGGAGG*TCTAGA**GCATCACTGACGCGTTGAGCC** Pru Gra10 KO 3’ FP

**GRA10PR2** *GTGAGCGGATAACAATTTCACACAGGAAACAGC*GCGGCCGC**CACGCTGTGTCGCAGACTCG**  Pru Gra10 KO 3’ RP

**GRA12PF1** *TTGGGTAACGCCAGGGTTTTCCCAGTCACGACG*GTTTAAAC**CCGACGACATCTTGGTCACACC** Pru Gra12 KO 5’ FP pRS416.GRA12P TGME49_288650 chrIX 2,657,360 to 2,659,155 (-)

**GRA12PR1** *GCGGGTTTGAATGCAAGGTTTCGTGCTGATCAA*ACTAGT**ACTGGCAGGCACTCGATAGGG** Pru Gra12 KO 5’ RP

**GRA12PF2** *TTCTGGCAGGCTACAGTGACACCGCGGTGGAGG*ACTAGT**GTTGGAGCAGCTCTTGCTCGAG** Pru Gra12 KO 3’ FP

**GRA12PR2** *GTGAGCGGATAACAATTTCACACAGGAAACAGCGCGGCCGC***CCATCTCCCATTGTTGAAAGAATGCG** Pru Gra12 KO 3’ RP

**GRA12TF1** *TTGGGTAACGCCAGGGTTTTCCCAGTCACGACG*GTTTAAAC**CCGACGACATCTTGGTCACACC** RH Gra12 KO 5’ FP pRS426.GRA12T TGGT1_288650 chrIX 2,468,014 to 2,469,809 (-)

**GRA12TR1** *GCGGGTTTGAATGCAAGGTTTCGTGCTGATCAA*ACTAGT**ACTGGCAGGCACTCGATAGGG** RH Gra12 KO 5’ RP

**GRA12TF2** *TTCTGGCAGGCTACAGTGACACCGCGGTGGAGG*ACTAGT**GTTGGAGCAGCTCTTGCTCGAG** RH Gra12 KO 3’ FP

**GRA12TR2** *GTGAGCGGATAACAATTTCACACAGGAAACAGCGCGGCCGC***CCATCTCCCATTGTTGAAAGAGTGCG** RH Gra12 KO 3’ RP

**GRA14PF1** *TTGGGTAACGCCAGGGTTTTCCCAGTCACGACG*GTTTAAAC**CTTCTAGCGAACACGTGGTAGCC** Pru Gra14 KO 5’ FP pRS416.pGRA14P TGME49_239740 chrVI 883,361 to 884,576 (-)

**GRA14PR1** *GCGGGTTTGAATGCAAGGTTTCGTGCTGATCAA*TCTAGA**GCTCGTTAGCGCGTCTCATGG** Pru Gra14 KO 5’ RP

**GRA14PF2** *TTCTGGCAGGCTACAGTGACACCGCGGTGGAGG*TCTAGA**ACTTCTGATGATATGCCCAGAGAGCC** Pru Gra14 KO 3’ FP

**GRA14PR2** *GTGAGCGGATAACAATTTCACACAGGAAACAGC*GCGGCCGC**CTCTTTGACAGAGGCACGGAACG** Pru Gra14 KO 3’ RP

**GRA15PF1** *TTGGGTAACGCCAGGGTTTTCCCAGTCACGACG*GTTTAAAC**CTCCACTGTATCGCATTGCAGGG** Pru Gra15 KO 5’ FP pRS416.pGRA15P TGME49_275470 chrX 7,286,502 to 7,290,563 (+)

**GRA15PR1** *GCGGGTTTGAATGCAAGGTTTCGTGCTGATCAAGCGGCCGC***CGTGTCTCAGTGCGACGCAC** Pru Gra15 KO 5’ RP

**GRA15PF2** *TTCTGGCAGGCTACAGTGACACCGCGGTGGAGGGCGGCCGC***GAAACCACCGATCCAGTGGACTC** Pru Gra15 KO 3’ FP

**GRA15PR2** *GTGAGCGGATAACAATTTCACACAGGAAACAGC*ACTAGT**AGTCCACGTGGTGGTCAGACC** Pru Gra15 KO 3’ RP

**Primers used for validation of genotype**

**5’DHFRCXR**  **ACTGCGAACAGCAGCAAGATCG** 5' integration validation RP for all KO's

**3’DHFRCXF** **GTTGGCCTACGTGACTTGCTGATG** 3' integration validation FP for all KO's

**GRA2PCXF** **GTTAACAGTTCCCTTGTGGCTGGTC** 5' integration validation FP

**GRA2PDF2** **GCTTCTTCTCCACATATCGCCTCAC** deletion validation FP

**GRA2PDR2 GGGAGTGGTGGTGTATGTTCACC** deletion validation RP

**GRA2PCXR CCCCAGGATAATGCAGAAAAGCTGG** 3' integration validation RP

**GRA3PCXF GCAAGGTCAACAGGGGTGCTTC** 5' integration validation FP

**GRA3PDF AATCAATCAGGCTCTTGCAAGAACCAG** deletion validation FP

**GRA3PDR2 GTCATCACTCGACGACGATAGGTAAG** deletion validation RP

**GRA3PCXR TCCGGACGTGTGTCCTGAGAG** 3' integration validation RP

**GRA7PCXF CAGGTGGGTCGAACAACCTCATTG** 5' integration validation FP

**GRA7PDF GATCACCTCACCACCAGCATGG** deletion validation FP

**GRA7PDR GAGTGCAGGCTGCCGTGATC** deletion validation RP

**GRA7PCXR**  **GCAGAAGAGGCCTGCGTGTC** 3' integration validation RP

**GRA8PCXF GCTTTGACATGGTAGATGCCCTGC** 5' integration validation FP

**GRA8PDF CTGCATGGAATGCCCAAGCCAG** deletion validation FP

**GRA8PDR GATTGCACCCCGCACGTGGAG** deletion validation RP

**GRA8PCXR GGCGATATCATCCAGGTGCGTG** 3' integration validation RP

**GRA9PCXF GAAACGCTGGCCTGTCCGTAC** 5' integration validation FP

**GRA9PDF GCCAGTACGTGGTTACTTGGGC** deletion validation FP

**GRA9PDR GACGTCACAGGGCTGGAATCC** deletion validation RP

**GRA9PCXR GCTGGTGGGTCTAATGCGCG** 3' integration validation RP

**GRA10PCXF CACGTGCTTTCCACCGTCGTG** 5' integration validation FP

**GRA10PDF CAGCAACTTCGGTCACACGCC** deletion validation FP

**GRA10PDR CAGTTGTACACGCAACTCCGGC** deletion validation RP

**GRA10PCXR TGCGGAGCATCGAGAGCTGG** 3' integration validation RP

**GRA12PCXF2 CTTCTGGTTCGGGCCAGCAC** Pru 5' integration validation FP

**GRA12TCXF GGCCAGCACAACTACTGAAGCTG** RH 5' integration validation FP

**GRA12DF CGATTTCGGGTGTACTTGTCAGCG** deletion validation FP

**GRA12DR ATGGAAGACCGTTTCTCTACAGGCTG** deletion validation RP

**GRA12CXR AGAGTTGAGTGCCTACGTCCCTC** 3' integration validation RP

**GRA14PCXF GGATAGACGTCCAAGACACACGG** 5' integration validation FP

**GRA14PDF**  **CTCCTCAGCAGCTTCATTGTGCC** deletion validation FP

**GRA14PDR GTCAACCGCCTCATGGAGACC** deletion validation RP

**GRA14PCXR AAATAGTCCTTCACTGTAGGTCGCAC** 3' integration validation RP

**GRA15PCXF**  **TGCCTCTAACACGCGTATGGTGTG** 5' integration validation FP

**GRA15PDF GTGCGCATTTGGGTGCTGTCC** deletion validation FP

**GRA15PDR CACGAACACCCCTTCAGACAAGC** deletion validation RP

**GRA15PCXR AGGCCCAAACTGGATCGAGGG** 3' integration validation RP

*Italicised nucleotides indicate regions of crossover in yeast recombination cloning, underlined nucleotides indicate restriction enzyme sites, and bold nucleotides indicate GRA12 specific priming targets (ToxoDB, version 27.0). FP and RP denote forward and reverse primers respectively.
