# Supplementary material for: Toxoplasma gondii Parasitophorous Vacuole Membrane-Associated Dense Granule Proteins Orchestrate Chronic Infection and GRA12 Underpins Resistance to Host Gamma Interferon
Source: mBio. 2019 Jul 2;10(4):e00589-19. doi: 10.1128/mBio.00589-19 (PMC6606796; doi:10.1128/mBio.00589-19)
Supplement: TABLE S3 [file mBio.00589-19-st003.docx]

**Table S3. Primers used to construct plasmids for complementation and validation of complemented GRA mutant strains.**

**Primer Sequence Primer Use Construct Corresponding locus**

**UPRPFF1** *TTGGGTAACGCCAGGGTTTTCCCAGTCACGACG*GTTTAAAC**GTGAGCTCATGCTGGAGCTTCG** UPRT 5’ Flank FP Pru UPRT vector flanks ME49_312480 chrXl 2,718,675 to 2,722,697 (+)

**UPRPFR1 AGCTTTCCGCTCGCTGGGAC** UPRT 5’ Flank RP GT1_312480 chrXI 2,681,773 to 2,685,796

**UPRPFF2** **GCCCTGCTGTCTTGTCAGGTACT** UPRT 3’ Flank FP

**UPRPFR2** *GTGAGCGGATAACAATTTCACACAGGAAACAGC*GCGGCCGC**CTGGCGTTCGATCGACCGAAG** UPRT 3’ Flank RP

**UPRGRA12PXF1A**  *CGTATTCCTTTTTTCGTCGGACCTTTCCACAGGGCTTCTA*AAAAGCTTACTAGT**GT** Pru GRA12 5’ FP pRS416.UPRGRA12_II_^FLHA^ ME49_288650 chrlX 2,657,228 to 2,659,728 (-) **AGCTGCCGCACCACGATG**

**UPRGRA12FLHAXR1N** *CGCGATTCCGTCAGCGGTCTGTCAAAAAAACTAGAGACC*GCTAGCCTAAGCAGCAG Pru GRA12 ^FLHA^ 3' RP

CGTAATCTGGAACATCGTATGGGTAGAGCTTGACTTTGTCATCGTCGTCCTTGTAGT

CGGCGCC**GTTGTGTTTGCTGCCTGCAGAGC**

**UPRNGRA12PXF** *CGTATTCCTTTTTTCGTCGGACCTTTCCACAGGGCTTCTAAAG*AAGCTTACTAGT**CCT** RH GRA12 5’ FP pRS416. UPNGRA12_I_^FLHA^ TGGT1_288650 chrlX 2,467,882 to 2,470,465 (-) **CGTCAGCGAAGCTGCCA**

**UPRGRA12FLHAXR1N** *CGCGATTCCGTCAGCGGTCTGTCAAAAAAACTAGAGACC*GCTAGCCTAAGCAGCAG RH GRA12^FLHA^ 3' RP

CGTAATCTGGAACATCGTATGGGTAGAGCTTGACTTTGTCATCGTCGTCCTTGTAGT

CGGCGCC**GTTGTGTTTGCTGCCTGCAGAGC**

**GRA12CRF** *CGTATTCCTTTTTTCGTCGGACCTGTCCACAGGGCTTCTAAAG*AAGCTTACTAGT**CCT** RH GRA12 FP pRS426. UPRGRA12_I_^HA^ TGGT1_288650 chrlX 2,467,882 to 2,470,465 (-)

**CGTCAGCGAAGCTGCCA**

**GRA12CRHAR** *CGCGATTCCGTCAGCGGTCTGTCAAAAAAACTAGAGACC*GTTTAAACTCAAGCGTAAT RH GRA12^HA^ RP

CTGGAACATCGTATGGGTA**GTTGTGTTTGCTGCCTGCAGAGC**

**Primers used for validation of genotype**

**UPRTCXF TCTCTCCCTGAGCTGCACGTG** 5' integration validation FP

**UGR12XR**  **CCCTCATCGTGTAGCAGATTGCG** 5' integration validation RP

**5'BGLIIGR12CL**  **AGCCATACGAGATCTCGAAGCCG** 3' integration validation FP

**UPRTCXR CCAGGTTCGACACTGGTCAGATG** 3' integration validation RP

**UPRTDF1 TGACGTCGGGTGCCTACGTTC** deletion validation FP

**UPRTDR1 CGACAGCTGCACTCGAAGACAC** deletion validation RP

*Italicised nucleotides indicate regions of crossover in yeast recombination cloning, underlined nucleotides indicate restriction enzyme sites, and bold nucleotides indicate priming regions specific to *Toxoplasma gondii* (ToxoDB, version 27.0). FP and RP denote forward and reverse primers respectively.
